# Neutrophil elastase as a versatile cleavage enzyme for activation of αvβ3 integrin-targeted small molecule drug conjugates with different payload classes in the tumor microenvironment

**DOI:** 10.3389/fphar.2024.1358393

**Published:** 2024-03-01

**Authors:** Anne-Sophie Rebstock, Mareike Wiedmann, Beatrix Stelte-Ludwig, Harvey Wong, Amy J. Johnson, Raquel Izumi, Ahmed Hamdy, Hans-Georg Lerchen

**Affiliations:** ^1^ Vincerx Pharma GmbH, Monheim, Germany; ^2^ Vincerx Pharma, Inc., Palo Alto, CA, United States

**Keywords:** αvβ3 integrin, tumor microenvironment, neutrophil elastase (NE), small molecule drug conjugate, camptothecin

## Abstract

**Introduction:** The development of bioconjugates for the targeted delivery of anticancer agents is gaining momentum after recent success of antibody drug conjugates (ADCs) in the clinic. Smaller format conjugates may have several advantages including better tumor penetration; however, cellular uptake and trafficking may be substantially different from ADCs. To fully leverage the potential of small molecule drug conjugates (SMDCs) with potent binding molecules mediating tumor homing, novel linker chemistries susceptible for efficient extracellular activation and payload release in the tumor microenvironment (TME) need to be explored.

**Methods:** We designed a novel class of SMDCs, which target αvβ3 integrins for tumor homing and are cleaved by neutrophil elastase (NE), a serine protease active in the TME. A peptidomimetic αvβ3 ligand was attached via optimized linkers composed of substrate peptide sequences of NE connected to different functional groups of various payload classes, such as camptothecins, monomethyl auristatin E, kinesin spindle protein inhibitors (KSPi) and cyclin-dependent kinase 9 inhibitors (CDK-9i).

**Results:** NE-mediated cleavage was found compatible with the diverse linker attachments via hindered ester bonds, amide bonds and sulfoximide bonds. Efficient and traceless release of the respective payloads was demonstrated in biochemical assays. The newly designed SMDCs were highly stable in buffer as well as in rat and human plasma. Cytotoxicity of the SMDCs in cancer cell lines was clearly dependent on NE. IC_50_ values were in the nanomolar or sub-nanomolar range across several cancer cell lines reaching similar potencies as compared to the respective payloads only in the presence of NE. *In vivo* pharmacokinetics evaluating SMDC and free payload exposures in rat and particularly the robust efficacy with good tolerability in triple negative breast and small cell lung cancer murine models demonstrate the utility of this approach for selective delivery of payloads to the tumor.

**Discussion:** These results highlight the broad scope of potential payloads and suitable conjugation chemistries paving the way for future SMDCs harnessing the safety features of targeted delivery approaches in combination with NE cleavage in the TME.

## 1 Introduction

Following Paul Ehrlich’s vision of a magic bullet (“Zauberkugel”), the development of bioconjugates for targeted delivery of anticancer agents is gaining momentum after recent success of ADCs in the clinics (Schwartz, 2004; Baah et al., 2021). Small molecule drug conjugates (SMDCs) may have several advantages, such as better tumor penetration, lack of immunogenicity and easier manufacturing (Cazzamalli et al., 2018; Zhuang et al., 2019). SMDCs may also differ from ADCs in cellular uptake and trafficking to the lysosomes; hence for SMDCs, alternatives to the traditional lysosomal payload release strategies successfully used for ADCs need to be found. To fully leverage the potential of SMDCs with potent binding molecules mediating efficient tumor homing, novel linker chemistries susceptible for efficient extracellular payload release in the TME need to be explored.

To improve tumor selectivity of cytotoxic agents and to enable treatment of aggressive cancers, we were particularly interested in SMDCs targeting αvβ3 integrins for efficient tumor homing. αvβ3 integrins are cell surface proteins, which are highly expressed on cancer cells and activated endothelial cells in a wide range of tumor types (Danen et al., 1994). The high expression is correlated with aggressive disease and poor prognosis for patients. These integrin receptors bind to arginine–glycine–aspartate (RGD)-containing matrix proteins and have an important function in tumor-induced angiogenesis. Inhibition of αvβ3 integrins has been extensively explored to inhibit angiogenesis and preclinical results looked promising (Hieken et al., 1996; Wu et al., 2013; Sun et al., 2019; Ludwig et al., 2021). In clinical trials integrin inhibitors were generally well tolerated, but unfortunately, due to crosstalk with VEGF receptors, no benefit in patients with cancer was observed with these agents (Hatley et al., 2018). Nonetheless, efficient, and selective tumor homing of integrin inhibitors has been shown in imaging studies and tissue analyses (Alday-Parejo et al., 2019). Therefore, a new focus of research was to use αvβ3 integrin ligands for delivery of cytotoxic payloads to cancer tissue (Korkmaz et al., 2008; Alloatti et al., 2012; Liu et al., 2012; Raposo Moreira Dias et al., 2019; Lerchen et al., 2022; Paulus and Sewald, 2022; Paulus et al., 2023).

**FIGURE 1:**
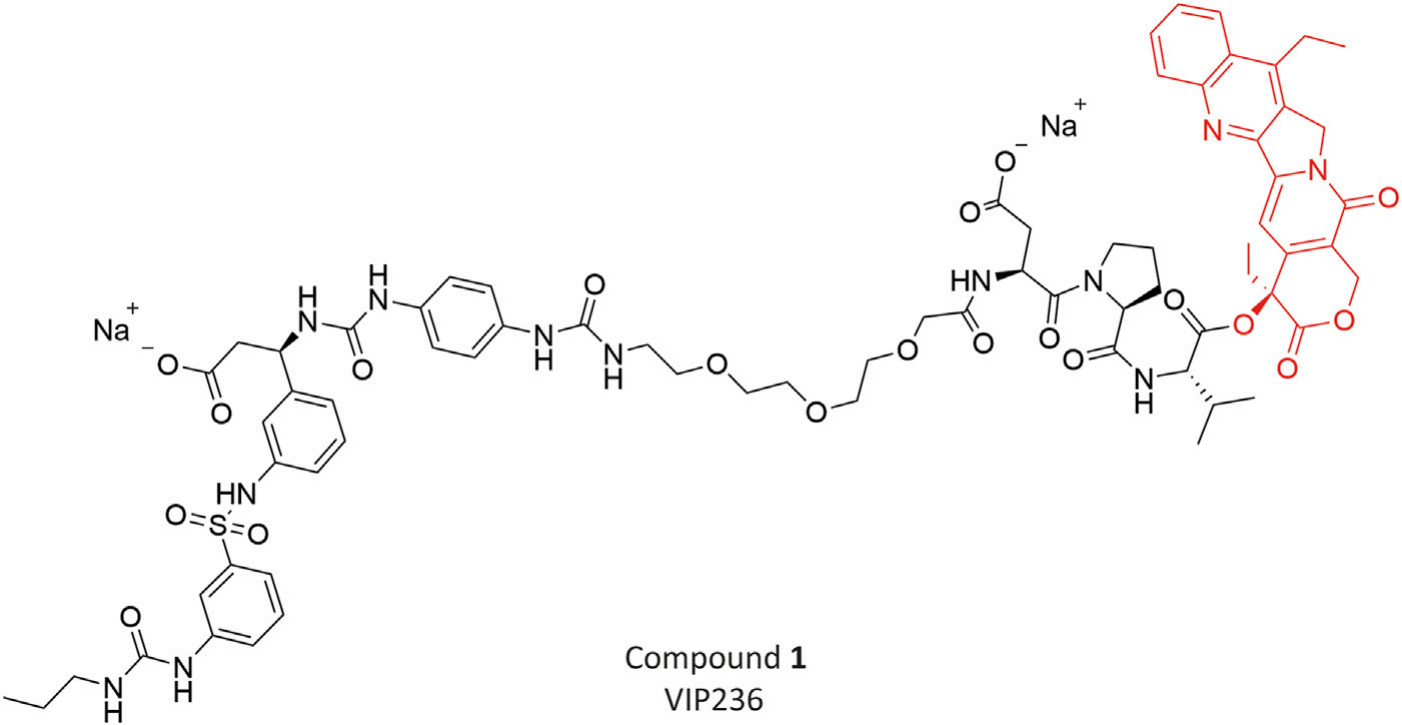
Chemical structure of Compound **1** (**VIP236**) with optimized CPT (Topoisomerase inhibitor) payload.

We previously described a novel class of SMDCs, which target αvβ3 integrins to deliver an optimized camptothecin payload (Lerchen et al., 2022). The peptidomimetic αvβ3 ligand employed in the conjugates was poorly internalized upon binding; therefore, we optimized the SMDCs for extracellular cleavage and payload release in the TME. We identified neutrophil elastase (NE), a serine protease active in the TME, as an appropriate enzyme to release the optimized camptothecin payload from the SMDC. Like αvβ3 integrins, the expression of NE is high in the stroma of many tumors, but low in normal tissues (Korkmaz et al., 2008; Raposo Moreira Dias et al., 2019) and its expression is associated with aggressive disease and metastasis (Sato et al., 2006; Lulla et al., 2023). Preclinical data of our lead compound **1** (**VIP236**) (Figure 1), which is currently in a Phase 1 clinical trial for treatment of advanced solid tumors (NCT05712889), have recently been published (Lerchen et al., 2023).

A particular challenge in the design and optimization of SMDCs is to achieve highly stable parent-molecules in circulation with efficient and selective cleavage only in TME. Here, we first investigated linker variations in the SMDC **1** (**VIP236)** and the impact on parent-molecule stability, release of 7-ethyl camptothecin payload and cytotoxic activity *in vitro* and *in vivo*. Compound **2** was selected for extended investigations as described in this paper. Another focus of this work was to broaden the scope and show the compatibility of NE-mediated activation of SMDCs with different payload classes with a particular emphasis on the cleavage site between linker and payload. We explored linker attachments to various functional groups on different payloads presenting a variety of potential cleavage sites for NE in SMDCs, such as hindered ester bonds, amide bonds and sulfoximide bonds. After the successful proof of concept with camptothecins, the peptidomimetic αvβ3 ligand was now attached via optimized linkers composed of substrate peptide sequences of NE to different functional groups of various payload classes, such as cyclin-dependent kinase 9 inhibitors (CDK-9i), monomethyl auristatin E (MMAE), and kinesin spindle protein inhibitors (KSPi). With each of these payloads presenting different linker attachment sites, the successful identification and characterization of lead SMDCs will be described showcasing the broad applicability of NE as versatile cleavage enzyme for a variety of drug conjugates. Stability data of respective SMDCs as well as the release of payloads in a traceless manner resulting in an NE-dependent *in vitro* cytotoxicity will be presented. Pharmacokinetic data in rat as well as *in vivo* efficacy data using xenograft models will also be shown.

## 2 Materials and methods

### 2.1 Compounds

The SMDC compounds **2**, **3**, **4**, **5**, and **6** were designed at Vincerx and synthesized in the Medicinal Chemistry Research Labs of Bayer AG, Pharmaceuticals, Wuppertal as outlined in the Supplementary Material.

### 2.2 Cell lines and *in vitro* proliferation assay in the presence and absence of neutrophil elastase

786-O (human RCC), HT-29 (human CRC), NCI-H292 (human NSCLC), NCI-H69 (human SCLC), MX-1 (human TNBC), and SUM149PT (human IBC) cells were acquired from ATCC and cultured according to the provider’s instructions.

The *in vitro* cytotoxicity of the SMDCs in comparison to the respective payloads was tested in a panel of cancer cell lines after a 72 h continuous exposure in presence or absence of 20 nM neutrophil elastase using MTT assays (ATCC). IC_50_ values were determined as the concentration of compound required for 50% inhibition of cell viability.

### 2.3 Stability assays in buffer at pH7.4 and in rat and human plasma

The SMDC compounds **2**, **3**, **4**, **5** and **6** (1 mg) were dissolved in acetonitrile/DMSO (1:1, 0.5 mL). While vortexing, 20 µL of this solution was added to 1 mL rat plasma at 37 °C. The incubation was stopped at respective time intervals by adding acetonitrile/buffer pH 3 (80:20) and the test compounds and formation of degradation products were analyzed by LC and LC/MS.

For hydrolytic stability measurement, compounds **2**, **3**, **4**, **5** and **6** (0.15 mg) were dissolved in acetonitrile/DMSO (4:1, 0.5 mL) and 1 mL of PBS buffer at pH 7.4 was then added. The sample solution was analyzed by HPLC to determine the amount of the test compound and the formation of degradation products over a period of 24 h at 37 °C.

### 2.4 Human neutrophil elastase cleavability assay

For the NE cleavability assay, the SMDC test compound was added to buffer (150 mM NaCl, 10 mM CaCl_2_, 0.05% BSA) to a final concentration of 5 µM (0.5% DMSO). The reaction was started by adding different concentrations of human NE (0, 20, 40, 60 nM) to the reaction vials. After incubation for 1 h at 37°C, the enzymatic reaction was stopped by precipitation in 50% ACN. Subsequently, the samples were subjected to HPLC-MS analysis to determine the concentration of test compound and of its metabolite.

### 2.5 Caco-2 cell permeability assays

The cell permeability of the payloads released by the SMDCs **5** and **6**, namely, compounds **10** and **11** were investigated with an *in vitro* flux assay using Caco-2 cells (Troutman and Thakker, 2003). The compounds were dissolved in HEPES buffer and applied (in triplicates) to the cells either on the apical (A) or basolateral (B) side of the Caco-2 monolayer at a final concentration of 2 μM. Before and after incubation for 2 h at 37 °C, samples were taken from both compartments and analyzed by LC-MS/MS. The apparent permeability coefficient (P_app_) was calculated for both the apical to basolateral (A → B) and the basolateral to apical (B → A) direction as described by Schwab et al. (Schwab et al., 2003).

### 2.6 *In Vivo* pharmacokinetics

Pharmacokinetic properties of SMDCs and their respective payloads were characterized in rats. Briefly, male Wistar rats (n = 3) were given a 0.5 mg/kg dose of SMDC formulated in plasma/DMSO (99:1) intravenously (i.v.) via tail vein. Blood was collected at pre-dose, 2, 5, 15, 30, 45, 60 min, 2, 4, 7, 24 h post-dose and then processed to plasma. Plasma was frozen at < −20°C until sample analysis. Plasma samples were analyzed for concentrations of SMDCs and their respective released payloads by LC-MS/MS.

In order to assess the selective concentration of payload to tumor tissue, the pharmacokinetic properties of the SMDC **3** and its payload **8**, in plasma and tumor was also characterized in female NMRI nu/nu xenograft mice (Taconic M&B A/S) bearing NCI H69 human small cell lung cancer tumors. The mice were treated i. v. via the tail vein with a single dose of **3** at 10 mg/kg. Plasma and tumor samples were collected at 0.25, 1, 7, and 24 h after treatment (*n* = 2 mice/group at each time point) and were stored frozen at < −20°C until sample analysis. Concentrations of the SMDCs and their respective payloads were measured by LC-MS/MS.

Pharmacokinetics of SMDCs and their respective payloads from *in vivo* studies were determined using mean concentration-time profiles. All pharmacokinetic parameters were calculated by non-compartmental methods as previously described (Perrier and Gibaldi, 1982).

### 2.7 *In vivo* efficacy in xenograft models

#### 2.7.1 MX-1 TNBC model

For *in vivo* efficacy studies, immunocompromised mice were inoculated subcutaneously with 1x10^6^ MX-1 TNBC cells in 50% Matrigel/50% Media on day 0. Treatment was started at a mean tumor volume of 75 mm^3^ in the MX-1 model (n = 12/group: Compound **2**, 20 mg/kg i. v. in a 2 days on/5 days off and in a once weekly (QW) schedule. **VIP236**, 20 mg/kg i. v. QW). Tumor and body weight were measured at least twice weekly.

#### 2.7.2 NCI H69 SCLC model

For the NCI H69 study, immunocompromised mice were inoculated subcutaneously with 1.3 × 10^6^ NCI H69 cells in 50% Matrigel/50% Media on day 0. Treatment was started at a mean tumor area in the range of 36–37 cm^2^ in the NCI H69 model (n = 8/group, compound **4** at 5 mg/kg i. v., QW, and compound **6** at 40 mg/kg i. v., QW). In an independent experiment in the same CDX xenograft model compound **3** was tested at 10 mg/kg i. v., QW. Tumor and body weight were measured at least twice weekly.

## 3 Results

### 3.1 Synthesis

**FIGURE 2:**
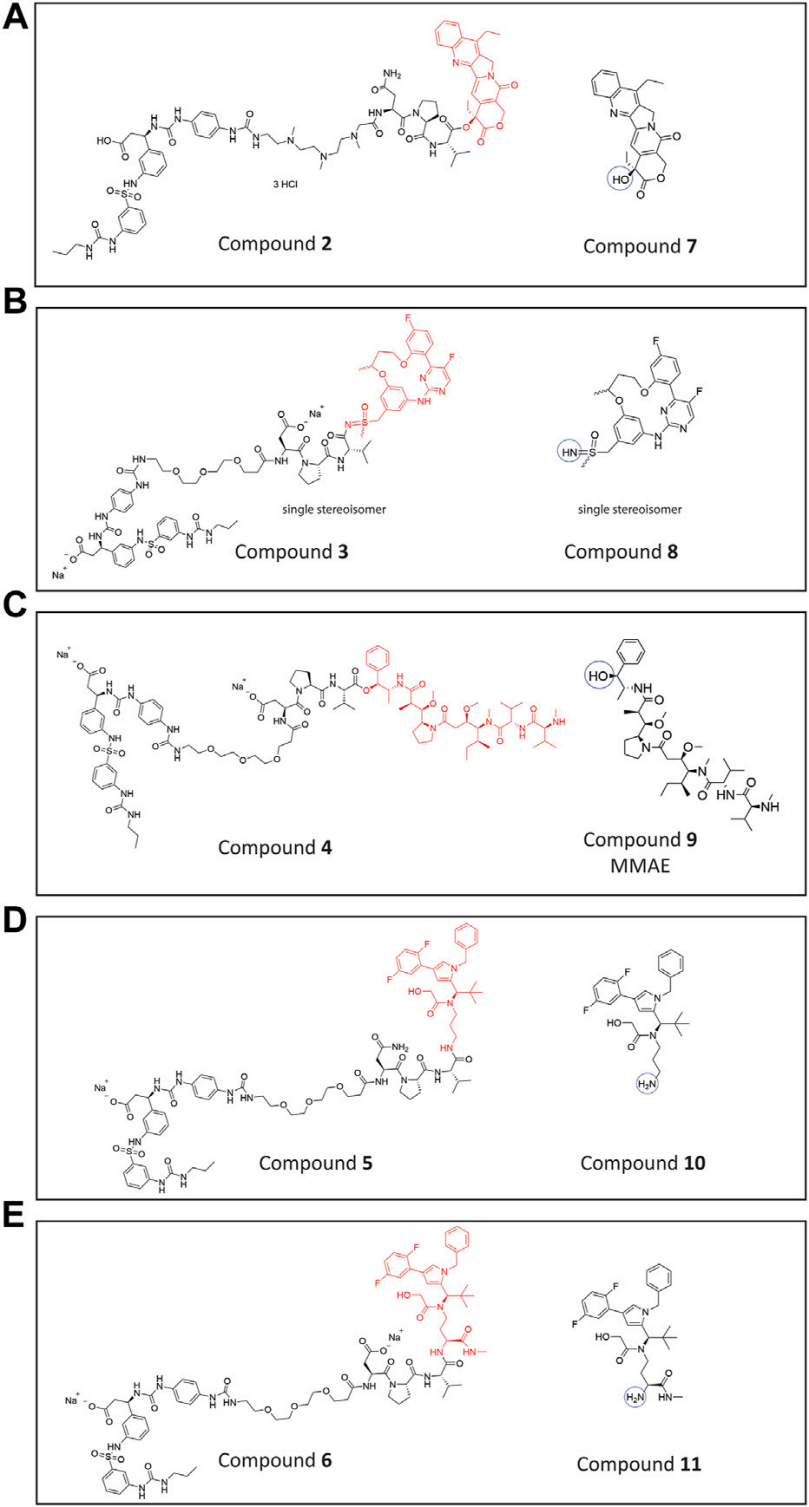
Chemical structures. **(A)** Conjugate **2** with 7-ethyl camptothecin (topoisomerase inhibitor) payload **7 (B).** Conjugate **3** with CDK-9 inhibitor payload **8 (C)**. Conjugate **4** with MMAE payload **9 (D)**. Conjugate **5** with KSPi (kinesin spindle protein inhibitors) payload **10 (E)**. Conjugate **6** with an optimized KSPi payload **11**.

The rationale of an initial study was, to replace the PEG linker in the lead compound **VIP236** by a poly-amine linker and the aspartic acid in the NE-cleavable substrate sequence with asparagine to obtain an overall basic SMDC, which may be retained in the acidic TME (Dharmaratne et al., 2021). Towards this goal, compound **2** (Figure 2A) with the same 7-ethyl camptothecin payload **7** (Kunimoto et al., 1987) but with a modified linker was synthesized in a similar way as previously described for **VIP236** (Lerchen et al., 2023) as outlined in the Supplementary Material.

Furthermore, we were striving for an expansion of the payloads compatible with NE-mediated activation. The peptidomimetic αvβ3 ligand was attached via optimized linkers composed of substrate peptide sequences of NE connected to different functional groups of various payload classes shown in Figures 2B–E, such as a CDK-9i **8** (Lücking et al., 2017), MMAE **9** (Singh, 2022) and KSPi **10** and **11** (Lerchen et al., 2018). In compound **3** (Figure 2B), the linker of **VIP236** is attached to a sulfoximine group in a CDK-9 inhibitor molecule **8** (Lücking et al., 2017) instead of the 20-hydroxy group in 7-ethyl camptothecin. This is to explore whether sulfoximides may also be recognized and cleaved by NE. The CDK-9i payload **8** employed in conjugate **3** is a single stereoisomer, isolated via chiral separation. The absolute configuration of **8** was not determined. The specific stereoisomer **8** was chosen as one of the two most potent isomers out of four based on *in vitro* cytotoxicity data (data not shown). In compound **4** (Figure 2C), a novel linker attachment to the sterically hindered hydroxy group of MMAE **9** is explored. In compounds **5** and **6** (Figures 2D, E) the linker is attached to an amino group present in KSPi molecules **10** and **11** which, as previously shown, is essential and needs to be liberated from the conjugates to provide an active metabolite (Lerchen et al., 2018).

The synthesis of these SMDCs is described in the Supplementary Material.

### 3.2 *In vitro* cytotoxicity

The cytotoxicity of the SMDCs was measured in different cancer cell lines in the absence and presence of NE and the results are shown in Table 1.

**TABLE 1:** Cytotoxicity *in vitro* in the absence and presence of elastase. IC_50_ (M) of SMDC compounds **2**, **3**, **4**, **5** and **6** in comparison to their corresponding payloads **7**, **8**, **9**, **10**, and **11**, respectively, in 4 cell lines with and without elastase.

Compound** **	786–0** **	786–0** ** + neutrophil elastase** **	HT29** **	HT29** ** + neutrophil elastase** **	NCI-H292** **	NCI-H292** ** + neutrophil elastase** **	SUM149PT** **	SUM149 PT** ** + neutrophil elastase** **
**2**	5.4E-7	3.5E-9	1.0E-6	2.0E-8	1.7E-7	6.0E-9	3.1E-7	4.8E-9
**7 **	3.7E-9	3.2E-9	2.2E-8	1.9E-8	3.8E-9	4.6E-9	3.8E-9	4.2E-9
**3 **	>1.0E-6	3.1E-9	9.9E-7	1.9E-9	8.8E-7	4.5E-9	7.7E-7	1.2E-9
**8 **	4.3E-9	4.9E-9	1.7E-9	1.3E-9	2.2E-9	2.2E-9	2.3E-9	2.6E-9
**4 **	6.6E-7	6.6E-9	7.8E-8	4.3E-10	7.1E-8	3.5E-10	5.0E-8	5.1E-10
**9 **	5.9E-9	4.9E-9	5.8E-10	5.5E-10	4.0E-10	3.6E-10	1.8E-10	1.4E-10
**5 **	1.0E-6	4.5E-9	1.0E-6	2.4E-9	9.9E-7	1.5E-9	3.3E-7	3.3E-10
**10 **	2.5E-9	2.5E-9	4.1E-10	4.1E-10	4.6E-10	2.7E-10	3.3E-10	1.7E-10
**6 **	1.0E-6	8.7E-9	1.0E-6	1.7E-9	1.0E-6	1.3E-9	1.0E-6	6.0E-10
**11 **	6.0E-9	6.0E-9	1.2E-9	1.4E-9	1.8E-9	9.7E-10	3.1E-10	5.3E-10

Cytotoxicity of SMDCs in cancer cell lines was clearly dependent on NE. All SMDCs (compounds **2**, **3**, **4**, **5** and **6**) had poor cytotoxicity after incubation for 72 h in culture medium without NE, whereas each of the five payloads (compounds **7**, **8**, **9**, **10** and **11**) was potent in the low nanomolar or even sub-nanomolar range. These results also indicate high stability and minimal payload release from the SMDCs under these conditions. However, when NE was added to the cell culture, cytotoxicity of the SMDCs increases significantly reaching IC_50_ values in the nanomolar or sub-nanomolar range across several cancer cell lines, thus, achieving similar potencies as compared with the respective payload alone. These results imply cleavage of the SMDCs, and release of the respective payloads mediated by NE.

### 3.3 Stability in buffer and in rat and human plasma

The newly designed SMDCs **2**, **3**, **4**, **5** and **6** were highly stable in buffer, in rat and in human plasma independent of the nature of the linkage of the cytotoxic payload to the NE-cleavable linker. Data are shown in the Supplementary Figure S1.

### 3.4 Cleavability by human neutrophil elastase

**FIGURE 3:**
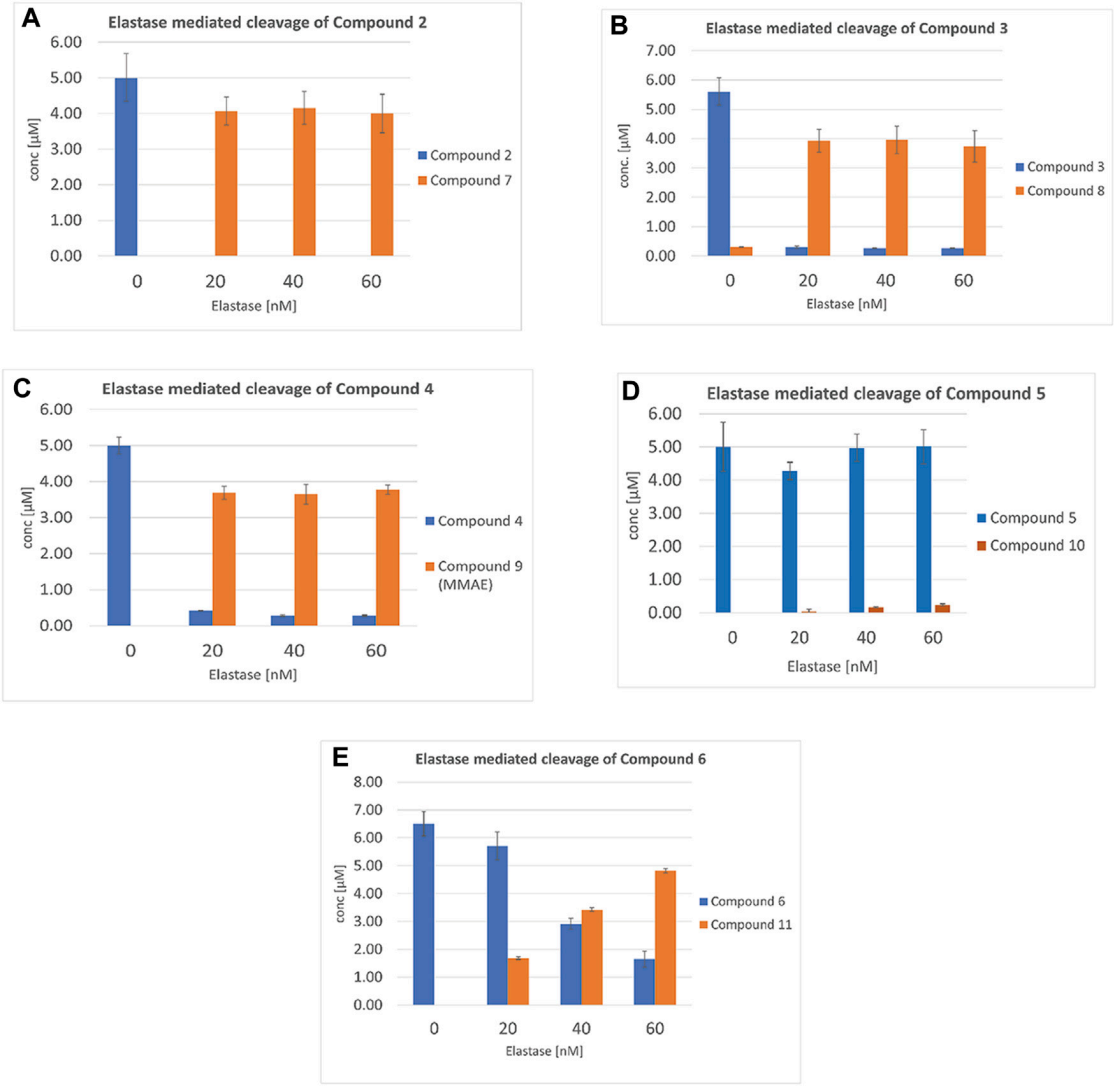
Neutrophil elastase cleavability assay of SMDCs: **(A)** Compound **2**, **(B)** Compound **3**, **(C)** Compound **4**, **(D)** Compound **5** and **(E)** Compound **6** monitoring concentrations of SMDC and released payloads **7**, **8**, **9**, **10** and **11**, with 20, 40 and 60 nM NE, respectively.

The initial design of elastase cleavable SMDCs such as **VIP236** was inspired by previous research on NE cleavable probes (Powers et al., 1977; Achilles and Bednarski, 2003). In our previous studies we have investigated **VIP236** and its epimers for NE cleavability showing that the natural L-configuration of the valine residue in position P1 of the identified NE-cleavable linker aspartic acid-proline-valine is crucial for efficient payload release (Lerchen et al., 2023). The new SMDCs **2**, **3**, **4**, **5** and **6** were investigated for cleavability by human NE in a biochemical assay at different NE concentrations. Conjugates **2**, **3** and **4** were essentially completely cleaved at all tested concentrations within 60 min (Figures 3A–C). The efficient NE cleavability of the SMDCs **VIP236** and **2** indicates that there is flexibility in position P3 and beyond as the negative charge of the aspartic acid in **VIP236** is not required and furthermore, a basic polyamine linker instead of a PEG linker is also tolerated. The efficient NE cleavability of SMDCs **3** and **4** is a striking finding demonstrating a broad substrate tolerance of NE particularly in the P1’–P3’ region. This includes the cleavage site itself which even tolerates a switch from an ester bond in compound **2** to a sulfoximide bond in compound **3** as well as entirely different ester substrates for NE presented in SMDCs **VIP236, 2** and **4**. The poor *in vitro* cytotoxicity of SMDCs **2**–**4** (Table 1) in the absence of NE demonstrates high stability of respective SMDCs requiring NE-mediated activation of the payload. For conjugate **5**, the cleavage was very slow and only small amounts of cleaved payload **10** could be detected after 60 min with the SMDC **5** remaining almost untouched. (Figure 3D). The conjugate **6** featuring an optimized KSPi payload **11** showed an improved NE-mediated cleavage increasing with higher NE concentrations (Figure 3E). These results indicate that the SAR of amide bonds to be cleaved by NE appears to be more restrictive as compared to ester bonds. Hence, NE-mediated cleavage of hindered ester bonds, amide bonds and sulfoximide bonds mediating efficient release of the respective payloads in a traceless manner was successfully demonstrated in biochemical assays.

### 3.5 Permeability in Caco-2 cells

Transporter pumps, such as the P-glycoprotein (P-gp) efflux pump expressed on cancer cells, are known to contribute to resistance to chemotherapy treatment (Seelig, 2020). Therefore, cellular permeability and the potential to resist efflux transporter pumps are important aspects in the selection of the antineoplastic agents to be used as the payload. The cytotoxic KSPi payloads **10** and **11** are released extracellularly from the SMDCs **5** and **6** with different kinetics. Here, we compared the permeability and efflux ratio of **10** and **11** using Caco-2 cells. **11** was approximately 10-times more permeable than **10** (P_app_ A-B). In addition, the efflux ratio of **11** was approximately 15 times lower than **10** in Caco-2, suggesting that the compound was transported out of the cells to a much lower extent (Table 2). These results suggest conjugate **6** is releasing a payload with a significantly improved *in vitro* profile compared with conjugate **5**.

**TABLE 2:** Permeability and efflux ratio of the payloads **10** and **11** measured in flux assays with Caco-2 cells. The values represent the mean value of at least two independent assays performed in triplicates (*n* = 3).

Compound	Caco-2 Flux P_app_ B-A	Caco-2 Flux​ P_app_ A-B	Caco-2 flux efflux ratio
10	179.7	3.8	129.1
11	303.0	34.8	8.7

### 3.6 *In vivo* pharmacokinetics

#### 3.6.1 Pharmacokinetic studies in rat

**FIGURE 4:**
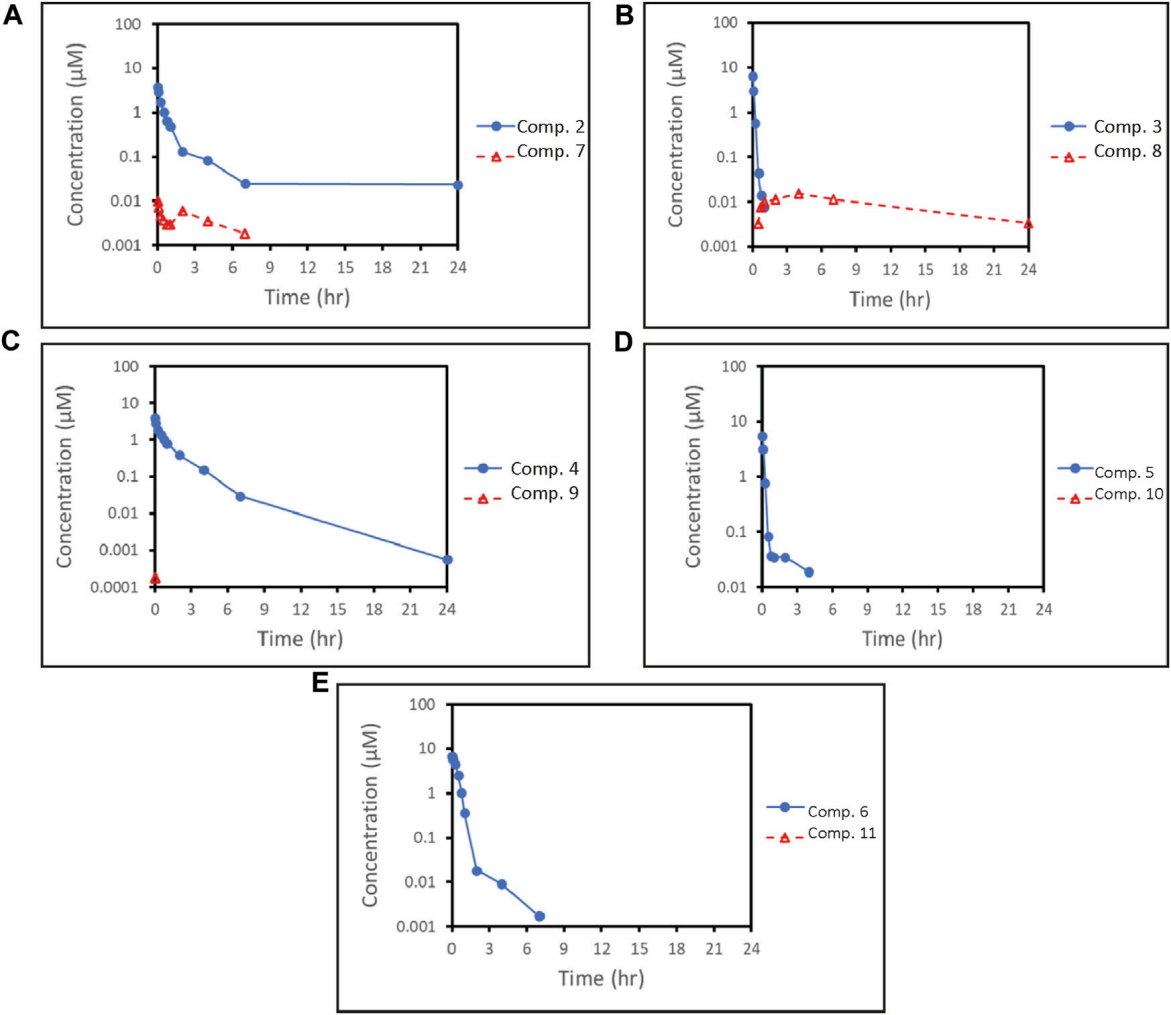
Plasma concentration-profiles of SMDCs (blue solid lines) Compound **2 (A)**, Compound **3 (B)**, Compound **4 (C)**, Compound **5 (D)** and Compound **6 (E)** and of their respectively released payloads (red dashed line) **7**, **8**, **9**, **10** and **11**. The concentrations of payloads **9 (C)**, **10 (D)** and **11 (E)** were largely below the lower limit of quantitation of the assay. Therefore, full plasma concentration-time profiles are not available for these payloads.

To compare the pharmacokinetic properties of the SMDCs, intravenous pharmacokinetic studies were performed in rat. Plasma concentration-time profiles of the SMDCs evaluated in the rat are shown in Figure 4 and pharmacokinetic parameters are shown in Table 3. Plasma clearance of all SMDCs evaluated was low in rats with clearance being in the following rank order: compound **3** > **5** > **2** > **6** > **4** (Table 3). Volume of distribution at steady state (Vss) was low for all SMDCs being less than total body water (<0.7 L/kg) (Davies and Morris, 1993). Specifically, for SMDCs **6** and **3**, the estimate of V_ss_ was comparable to plasma volume. The half-life (t_1/2_) for each SMDC tended to rank in order of their respective V_ss_ estimates. The release of payload in rat plasma was low for all SMDCs evaluated with ratio of payload to parent SMDC AUC on a molar basis ≤0.0105. The exception was compound **3** where the ratio of payload to parent SMDC AUC was ∼0.31.

**TABLE 3:** Pharmacokinetic parameters of conjugates **2**, **3**, **4**, **5** and **6** and release of their respective payloads.

Parameter	Compound				
	2	3	4	5	6
CL_p_ (mL/min/kg)	2.33 ± 0.44	6.60 ± 0.95	1.47 ± 0.06	5.50 ± 2.35	1.69 ± 0.15
V_ss_ (L/kg)	0.627 ± 0.344	0.0366 ± 0.0053	0.157 ± 0.015	0.600 ± 0.515	0.0377 ± 0.0050
t_1/2_ (h)	8.31 ± 5.49	0.240 ± 0.050	2.30 ± 0.95	4.88 ± 4.37	0.442 ± 0.023
AUC_0-inf_ (µmol*hr/L)	2.52 ± 0.61	0.801 ± 0.126	3.03 ± 0.11	1.09 ± 0.49	2.98 ± 0.25
payload/parent AUC ratio	0.0105	0.307	NC	NC	NC

AUC, area under the plasma concentration-time profile; AUC0-inf, area under the plasma concentration-time profile from time 0 to infinity; Clp, plasma clearance; t1/2, elimination half-life; Vss, volume of distribution at steady-state; NC, not calculated as warhead not detected (LLOQ < 0.0005 µM).

### 3.7 *In vivo* efficacy

#### 3.7.1 Investigation of SMDC linker variations

**FIGURE 5:**
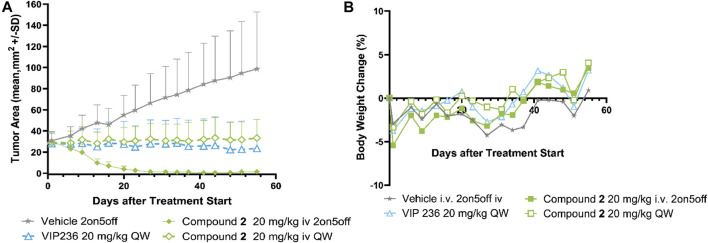
Efficacy of compound **2** compared to **VIP236** in TNBC MX-1 CDX model. **(A)** Growth curve of MX-1 tumors treated with compound **2** in two schedules (2 days on/5 days off schedule and once weekly) and **VIP236** with once weekly dosing. **(B)** Body weight changes based on compound **2** and **VIP236** treatment are minor. All treatment groups show statistical significance *p* < 0.0001 vs. vehicle (unpaired *t*-test).

We sought to investigate the effect of varying the linker in the SMDC **VIP236**, which resulted in compound **2** with a basic poly-amine linker instead of the PEG linker used in **VIP236**. Compound **2** with the modified linker was investigated in comparison to the lead **VIP236** in the MX-1 TNBC xenograft model. Results are shown in Figures 5A, B.

Monotherapy of compound **2** demonstrated significant tumor regression in the 20 mg/kg 2on/5off schedule and was well tolerated as no significant body weight loss was observed. At a once weekly dosing of 20 mg/kg of compound **2** tumor stasis could be achieved, which was comparable to **VIP236** (Figure 5A). Based on body weight changes both compounds showed good tolerability (Figure 5B).

### 3.7.2 Broadening of the payload scope compatible with NE-mediated cleavage

We also sought to investigate a broadening of the payload scope and identified three different payload classes compatible with NE-mediated cleavage *in vitro*. The new SMDCs featuring a CDK-9i, MMAE, or KSPi payloads were investigated in an NCI H69 SCLC xenograft model. Initially, the SMDC **3** with the CDK-9i payload **8** was tested in the NCI H69 SCLC model with a weekly dose of 10 mg/kg in 3 treatment cycles. No significant inhibition of NCI-H69 tumor growth could be demonstrated with this dose and schedule (data not shown).

**FIGURE 6:**
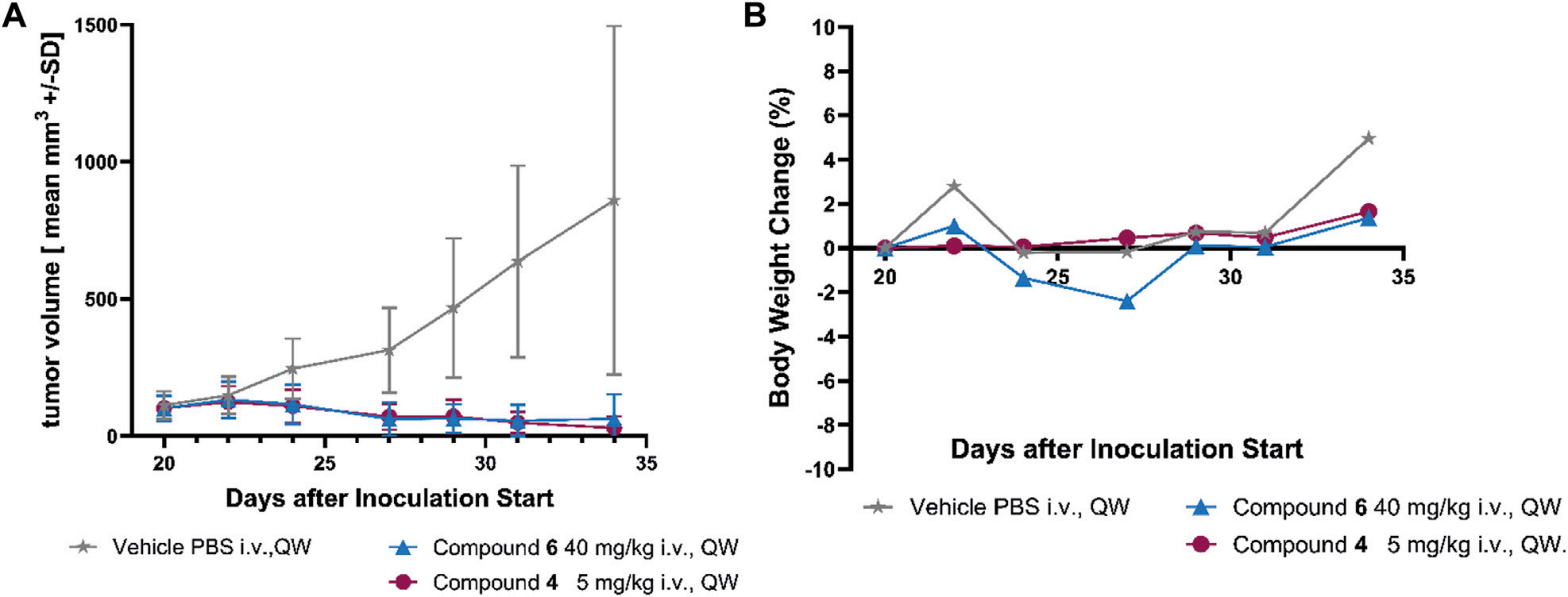
Efficacy of compound **4** and compound **6** in the SCLC NCI H69 xenograft model. **(A)** Growth curves of NCI H69 tumors treated with compound **4** and compound **6** once weekly for two cycles (QWx2). Monotherapy of **4** and **6** achieved significant tumor regression. **(B)** Body weight changes based on compound **4** and compound **6** monotherapies are very minor. All treatment groups show statistical significance *p* < 0.01 vs. vehicle (unpaired *t*-test).

In a subsequent experiment the MMAE SMDC compound **4** and the optimized KSPi SMDC compound **6** were also evaluated in the NCI H69 xenograft model. The results are shown in Figures 6A, B.

Monotherapy of SMDC **4** with MMAE as payload achieved statistically significant tumor regression with two applications of 5 mg/kg i. v. once weekly. Also, treatment with compound **6** with optimized KSPi payload at a dose of 40 mg/kg i. v. demonstrated statistically significant tumor regression with the same schedule (Figure 6A). The dosages were selected based on a previous MTD study (data not shown). Both therapies showed a high tolerability based on the body weight changes shown in Figure 6B.

**FIGURE 7:**
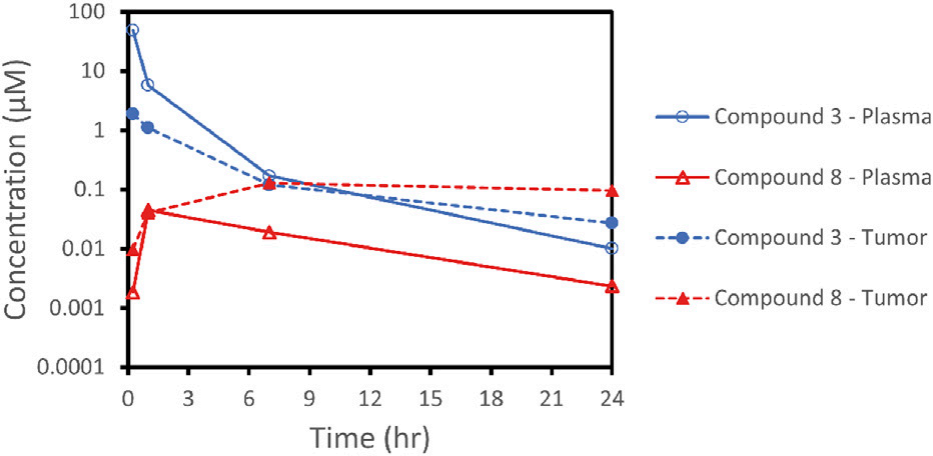
Plasma and tumor concentration-profiles of the CDK-9i SMDC **3** and the released payload **8**.

To further investigate the finding that SMDC **3** was inactive *in vivo* and to verify that delivery of SMDCs resulted in concentration of the released payload in tumor tissue as previously demonstrated for **VIP236** (Lerchen et al., 2022), a pharmacokinetic study was performed in NCI H69 tumor-bearing mice. After i. v. administration of 10 mg/kg of compound **3**, plasma and tumor concentrations of the SMDC **3** and of the released payload **8** were measured; the results are shown in Figure 7 and in Table 4. The exposure of the released payload **8** was 7.6-fold higher in the tumor *versus* plasma, indicating a successful delivery of the free payload to tumor tissue (Table 4). Despite this nice accumulation of the CDK-9i payload **8** in tumor over plasma shown in the PK study, tumor exposures of **8** appear not to be sufficient to significantly inhibit tumor growth. Further optimization is ongoing with the goal to also demonstrate the proof of *in vivo* efficacy of SMDCs with sulfoximide-linked CDK-9i payloads for treatment of solid tumors.

**TABLE 4:** Exposures (C_max_ and AUC_0-t_) of conjugate **3** and its respective payload **8** in plasma and tumor/plasma ratio in NCI H69 tumor bearing mice.

PK parameters	Compound 3 10 mg/kg IV	
	Conjugate 3	Payload 8
C_max_ (µM)	49	0.045
AUC_0-t_ (µmol*hr/L)	43	0.33
tumor/plasma AUC ratio	0.126	7.58

AUC, area under the plasma concentration-time profile; AUC_0-t_, area under the plasma concentration-time profile from 0 to the last timepoint; C_max_,maximum concentration observed. LLOQ, is 0.001 µM.

## 4 Discussion

SMDCs are currently gaining momentum with potential advantages over ADCs, such as better tumor penetration and lack of immunogenicity (Cazzamalli et al., 2018). For the treatment of aggressive cancers, we are developing SMDCs using a peptidomimetic αvβ3 integrin binder, which shows efficient tumor homing, and a linker, which is extracellularly cleaved by NE in the TME (Lerchen et al., 2022). Our lead SMDC **VIP236** (Lerchen et al., 2023) is currently in a Phase 1 clinical trial for the treatment of advanced solid tumors (NCT05712889). Preclinically, **VIP236** showed optimal activity when administered with a 2 days on/5 days off therapy schedule. Here, we show our investigations on linker variations as well as our successful efforts to expand the payload scope in αvβ3 targeted SMDCs, which are susceptible to cleavage by NE. Pharmacokinetic studies in rat suggest that low clearance of the SMDC can still be maintained with changes in the payload class and linker. In addition, for most of the evaluated SMDCs, the payload/parent AUC ratio is low, suggesting stability of the linker and low systemic exposure to the payload.

### 4.1 Linker variation

We first explored linker variations and designed a **VIP236** analogue with the goal to improve retention in the acidic environment of the TME to potentially increase efficacy with once weekly dosing. SMDC **2** uses the same 7-ethyl camptothecin payload **7** (Kunimoto et al., 1987) as **VIP236**; however, the PEG linker present in the lead compound **VIP236** was replaced by a poly-amine linker and the aspartic acid was replaced by asparagine to obtain an overall basic SMDC, which may increase retention in the acidic TME as the SMDC will be in its protonated form (Dharmaratne et al., 2021). The *in vivo* results shown in the MX-1 TNBC model demonstrate, that **2** is a highly potent SMDC achieving complete tumor regression with a dose of 20 mg/kg administered in a 2 days on/5 days off schedule, like also previously shown with **VIP236** at a dose of 26 mg/kg in a 3 days on/5 days off schedule (Lerchen et al., 2022; Lerchen et al., 2023). At a once weekly dosing of 20 mg/kg of SMDC **2** tumor stasis could be achieved, which was comparable to **VIP236** (Figure 5A). In conclusion, the basic poly-amine linker is an alternative to the PEG linker used in **VIP236** that further broadens the modular toolbox for SMDCs.

### 4.2 Extension of payload scope

In another aspect of this work, we were striving for an expansion of the payload scope compatible with NE-mediated activation. Our focus was to investigate whether functional groups present on different payloads could be compatible with the attachment of linkers. The resulting linkage should be efficiently cleaved by NE and allow for traceless release of the payload. At the same time corresponding SMDCs should be highly stable in circulation. Raposo Moreira Dias et al. (Raposo Moreira Dias et al., 2019) described SMDCs releasing the paclitaxel payload via NE-mediated cleavage, which however, required two subsequent steps of first fragmentation and then cyclization to release the payload. The goal of our work was to use NE cleavage for direct and ‘traceless’ release of the respective payload avoiding further conversion steps of intermediates with different clearances. This approach may reduce complexity of DMPK and increase the efficiency of payload formation.

### 4.3 Monomethyl auristatin E

Protease-cleavable SMDCs using the clinically validated MMAE payload from vedotin ADCs (Senter and Sievers, 2012), with substrate sequences for cathepsin B (Bennett et al., 2020; Davis et al., 2023; Zambra et al., 2023) or legumain (Liu et al., 2012) all depend on a self-immolative para-amidobenzyloxy carbamate (PABC) moiety (Carl et al., 1981) to release the active payload MMAE. To overcome shortcomings associated with the increased hydrophobicity of such self-immolative PABC linker approaches, we were looking for alternative linker attachments enabling a traceless payload release. Due to our previous experience with NE-cleavable linkers attached to camptothecins via sterically hindered ester bonds, we investigated the hydroxy group of the nor-ephedrin amino acid present in MMAE for its suitability as a conjugation site for NE-cleavable linkers without the need for self-immolative moieties. The high stability of the ester-linked MMAE SMDC **4** *in vitro* reported in Section 3.3 nicely fits to the low levels of free payload detected in the PK studies of the SMDC in rat (Section 3.6). This stability profile in combination with the efficient and specific cleavability by NE and consequently the NE-dependent cytotoxicity of **4** demonstrate the utility of this new linker attachment site in MMAE bioconjugates to enable traceless release of the payload. Based on this profile with extracellular payload release specifically mediated by NE in a traceless manner and the high activity observed in the NCI H69 xenograft model with only two doses of 5 mg/kg administered in a once weekly schedule, the SMDC **4** represents a novel and promising option to deliver MMAE to aggressive cancers. This SMDC **4** combines excellent stability *in vivo* with high efficacy upon specific NE-mediated release of the MMAE payload **9** in TME. Further investigations of dose and schedule as well as characterization of safety, ADME properties and efficacy in patient-derived tumor models are warranted.

### 4.4 CDK-9i

Another payload class which attracted our interest was inhibitors of CDK-9. Advanced clinical compounds such as atuveciclib (Lücking et al., 2017) and enitociclib (Lücking et al., 2021) have shown promising activity for the treatment of hematological malignancies. We envisioned the sulfoximine group present in CDK-9i, such as atuveciclib and enitociclib, as potential linker attachment site. The successful conversion of sulfoximines to pseudopeptides was described previously (Bolm et al., 1997). We chose to investigate the sulfoximines as the attachment site for NE-cleavable substrate peptides and their utility as part of NE-cleavable SMDCs. To explore treatment options also for solid tumors with αvß3-targeted CDK-9i SMDCs, we considered a highly potent subclass of macrocyclic CDK-9i such as **8**, which retains the atuveciclib core. Synthesis of the αvß3-targeted SMDC **3** was successfully accomplished, and this SMDC, with a sulfoximide bond as linkage between payload and linker, met our criteria for efficient NE-mediated cleavage, NE-dependent cytotoxicity as well as high stability in plasma. In comparison to the other SMDCs described here, compound **3** showed a higher ratio of released payload compared with the parent SMDC in pharmacokinetic studies in rat as described in Section 3.6. When pharmacokinetic studies were performed in NCI H69 tumor bearing mice, a 7.6-fold higher exposure of released payload **8** was detected in the tumor *versus* plasma, indicating a successful delivery of the free payload to tumor tissue. These results validate the use of a sulfoximide linker as a new NE-cleavable functionality in drug conjugates. Further optimization is ongoing to demonstrate an *in vivo* proof of sulfoximide-linked CDK-9i as payloads in SMDCs for treatment of solid tumors.

### 4.5 KSPi

Kinesin spindle protein inhibitors (KSPi) have been successfully employed as payloads in ADCs, particularly when releasing a non-permeable metabolite modified with a CellTrapper moiety enabling tumor accumulation after intracellular cleavage (Kirchhoff et al., 2020). For utilization of KSPi payloads in SMDCs with extracellular cleavage, the physicochemical profile of the KSPi needs to be tuned for a different profile with high potency and a higher membrane permeability. The amino group present in KSPis may allow for the attachment of substrate sequences potentially cleaved by NE. Cleavability by NE would be a requirement, as this amino group has previously been shown to be essential for activity (Lerchen et al., 2018). The initially identified KSPi payload **10** was highly potent, but it also showed a high active efflux (Table 1 and 2). On top of this, the corresponding amide linked SMDC **5** showed only very slow and inefficient release of the payload **10** in the biochemical NE cleavage assay (Figure 3). Interestingly, inserting an N-methyl carboxamide moiety next to the amino group resulted in KSPi **11**, which was equipotent to **10** (Table 1) but had a significantly improved physicochemical profile with higher permeability and reduced efflux (Section. 3.5; Table 2). The corresponding SMDC **6** with a NE-cleavable linker attached to the amino group of the payload **11** also showed a significantly improved cleavability by NE *versus* **5**. Consequently, this optimized SMDC **6** was highly efficacious and well tolerated in the NCI H69 xenograft model with two doses of 40 mg/kg given i. v. once weekly. This initial data confirms successful delivery of KSPi to tumors with potentially less frequent dosing schedules than required for small molecule KSPis, such as ispinesib (Purcell et al., 2010). Furthermore, the lack of exposure of systemically cleaved payload **11** after i. v. administration of the SMDC **6** to rat (Figure 4E) and no significant body weight loss observed in the *in vivo* studies demonstrate the potential of SMDCs with KSPi payloads such as **6** for high efficacy with lower side effects.

## 5 Conclusion

We have presented three case studies of αvß3-targeted SMDCs, **2**, **4** and **6**, which demonstrate the versatile utility of NE-cleavable linkers in SMDCs. High stability of the SMDCs *in vitro* and *in vivo* combined with efficient cleavage by NE resulted in high NE-dependent cytotoxicity. Initial efficacy data in xenograft models showed tumor regression with good tolerability demonstrating the utility of this approach for selective delivery of payloads to the TME. These studies highlight the broader scope of potential payloads and tolerated conjugation chemistries paving the way for future SMDCs harnessing the safety features of αvß3-targeted delivery approaches in combination with NE-mediated cleavage in the TME.

## Data Availability

The original contributions presented in the study are included in the article/Supplementary Material, further inquiries can be directed to the corresponding author.
