# Supplementary material for: Neutrophil elastase as a versatile cleavage enzyme for activation of αvβ3 integrin-targeted small molecule drug conjugates with different payload classes in the tumor microenvironment
Source: Front Pharmacol. 2024 Mar 1;15:1358393. doi: 10.3389/fphar.2024.1358393 (PMC10943695; doi:10.3389/fphar.2024.1358393)
Supplement: Supplementary file 1 [file DataSheet1.pdf]

Neutrophil Elastase as a Versatile Cleavage Enzyme for Activation of αvβ3 Integrin-Targeted Small-Molecule-Drug-Conjugates with Different Payload Classes in the Tumor Microenvironment

Anne-Sophie Rebstock[1], Mareike Wiedmann[1], Beatrix Stelte-Ludwig[1], Harvey Wong[2], Amy J. Johnson[2], Raquel Izumi[2], Ahmed Hamdy[2] and Hans-Georg Lerchen[1*]

Supplementary data

1 Synthesis of SMDCs 1
General Procedures 1
Synthesis of Compound 2 2
Synthesis of Compound 3 3
Synthesis of Compound 4 4
Synthesis of Compound 5 10
Synthesis of Compound 6 16
2 Supplementary figures 22
Figure S1: Rat plasma, human plasma, and buffer pH 7.4 stabilities of SMDCs 22

Supplementary data

1 Synthesis of SMDCs

General Procedures

All commercial reagents and catalysts were used as provided by the commercial supplier without purification. Solvents for synthesis, extraction and chromatography were of reagent grade and used as received. Moisture-sensitive reactions were carried out under an atmosphere of argon, and anhydrous solvents were used as provided by the commercial supplier. ^{1}H NMR and ^{13}C NMR spectra were recorded at room temperature (RT) with Bruker Avance spectrometers. Chemical shifts (δ) are reported in ppm relative to TMS as an internal standard.

The descriptions of the coupling patterns of 1H NMR signals are based on the optical appearance of the signals and do not necessarily reflect the physically correct interpretation. In general, the chemical shift information refers to the center of the signal. In the case of multiplets, intervals are given.

Analytical mass spectrometry was performed on HPLC/MS (Waters, Agilent, Thermo Fisher) using Waters Time-of-Flight, Waters/Micromass Single Quadrupole, or Thermo Fisher Scientific Orbitrap mass spectrometers. Ionization methods were electrospray ionization (ESI) positive/negative or electron ionization (EI).

LC/MS analyses were performed using the respective method as noted.

Method 1:

System MS: Thermo Scientific FT-MS; System UHPLC+: Thermo Scientific Vanquish; Column: Waters, HSST3, 2.1 x 75 mm, C18 1.8 µm; Eluent A: 1 l Water + 0.01% Formic acid; Eluent B: 1 l Acetonitrile + 0.01% Formic acid; Gradient: 0.0 min 10% B → 2.5 min 95% B → 3.5 min 95% B; Oven: 50°C; Flow: 0.90 ml/min; UV-Detection: 210 nm.

Method 2:

System MS: Waters TOF instrument; System UPLC: Waters Acquity I-CLASS; Column: Waters, HSST3, 2.1 x 50 mm, C18 1.8 µm; Eluent A: 1 l Water + 0.01% Formic acid; Eluent B: 1 l Acetonitrile + 0.01% Formic acid; Gradient: 0.0 min 2% B → 0.5 min 2% B → 7.5 min 95% B → 10.0 min 95% B; Oven: 50°C; Flow: 1.00 ml/min; UV-Detection: 210 nm.

Method 3:

System MS: Thermo Scientific FT-MS; System UHPLC+: Thermo Scientific UltiMate 3000; Column: Waters, HSST3, 2.1 x 75 mm, C18 1.8 µm; Eluent A: 1 l Water + 0.01% Formic acid; Eluent B: 1 l Acetonitrile + 0.01% Formic acid; Gradient: 0.0 min 10% B → 2.5 min 95% B → 3.5 min 95% B; Oven: 50°C; Flow: 0.90 ml/min; UV-Detection: 210 nm/ Optimum Integration Path 210-300 nm.

Synthesis of Compound 2

The synthesis of Compound 2 has been described in WO2023/057813 as Compound B4b according to the scheme depicted below.

This is a provisional file, not the final typeset article

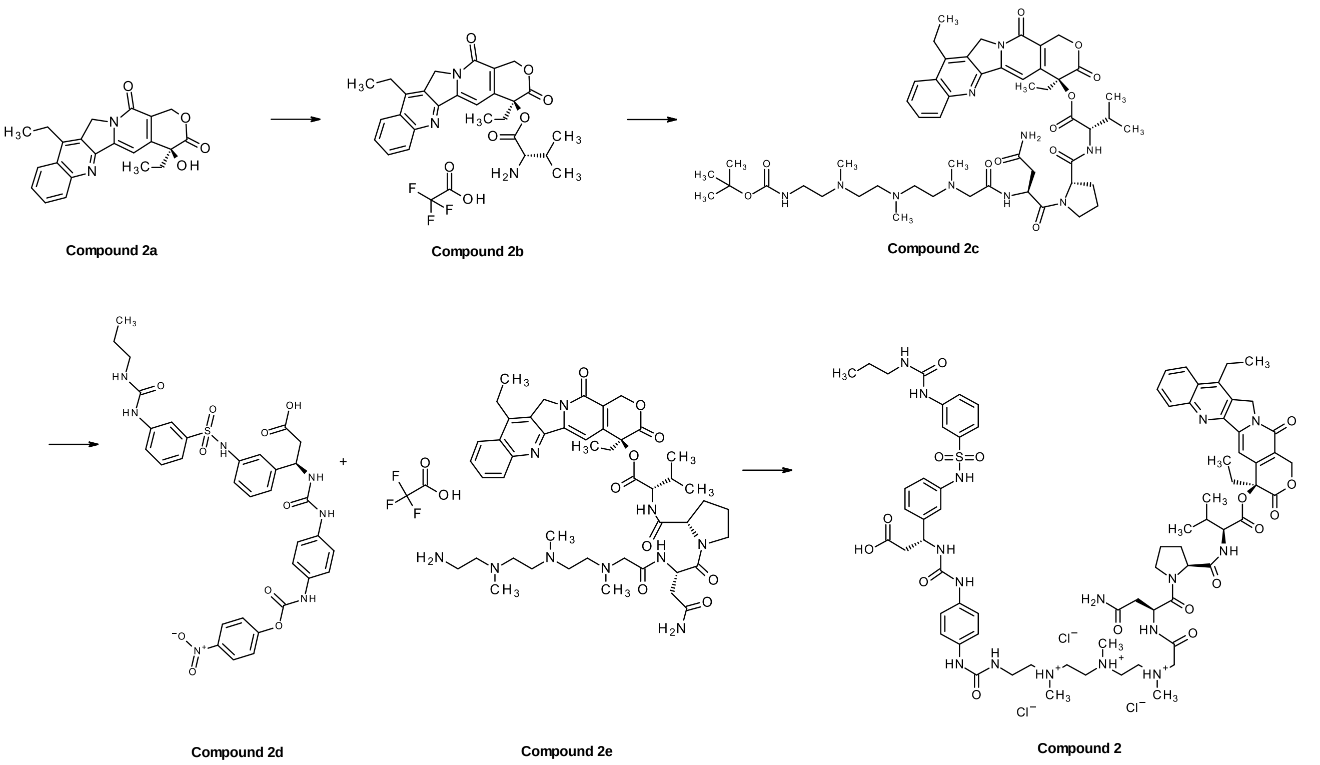

Analytical data of Compound 2:

LC-MS: R_t = 2.92 min; MS (ESIpos): m/z = 1495 $[M+H]^+$.

^{1}H-NMR (600 MHz, DMSO-d6): d [ppm] = 10.27 (s, 1H), 9.00 (s, 2H), 8.78 (s, 1H), 8.49 (s, 1H), 8.29 (t, 2H), 8.04 (s, 1H), 8.03 (d, 2H), 7.86 (t, 1H), 7.73 (t, 1H), 7.57 (br s, 1H), 7.43 (br d, 1H), 7.28-7.21 (m, 7H), 7.17-7.13 (m, 2H), 7.04 (br s, 1H), 7.01-6.93 (m, 2H), 6.89 (d, 1H), 6.73 (br d, 1H), 5.49 (s, 2H), 5.34 (br d, 2H), 5.00 (br d, 2H), 4.07 (t, 2H), 3.77 (br t, 4H), 3.63-3.56 (m, 6H), 3.34-3.28 (m, 3H), 3.28-3.14 (m, 7H), 3.13-2.94 (m, 5H), 2.85 (s, 4H), 2.80 (br s, 4H), 2.72-2.59 (m, 4H), 2.55-2.52 (m, 9H), 2.49-2.35 (m, 3H), 2.25-2.13 (m, 5H), 1.96 (br d, 3H), 1.46-1.38 (m, 2H), 1.31 (t, 4H), 0.98-0.84 (m, 13H).

Synthesis of Compound 3

The synthesis of Compound 3 has been described in WO2023/057812 A1 as Example C48 according to the scheme depicted below. The synthesis of Compound 3a is described in the same patent application as Building Block 18.

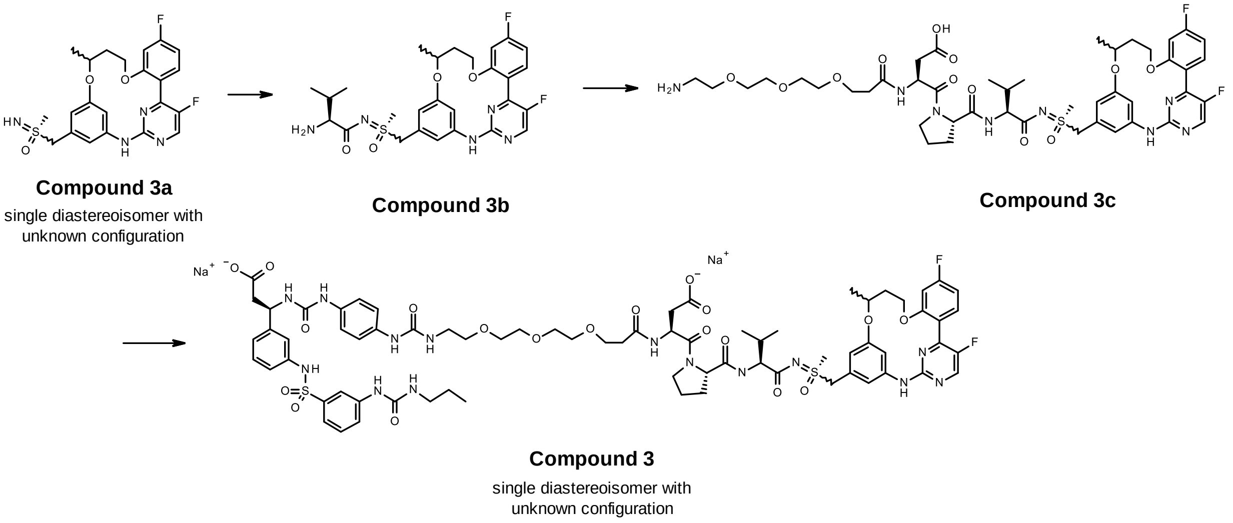

Analytical data of Compound 3:

LC-MS (Method 3): R_t = 4.43 min; MS (ESIpos): m/z = 1554 $[M+H]^+$.

^{1}H-NMR (600 MHz, DMSO-d6) δ [ppm]: 9.91 (br d, 1H), 9.75 (s, 1H), 8.79 (br s, 1H), 8.76-8.67 (m, 2H), 8.30-8.20 (m, 1H), 8.16-8.09 (m, 1H), 7.85-7.76 (m, 2H), 7.65-7.58 (m, 1H), 7.58-7.54 (m, 1H), 7.38-7.32 (m, 1H), 7.31-7.27 (m, 2H), 7.23 (q, 4H), 7.18 (d, 1H), 7.07 (t, 1H), 6.94 (br d, 1H), 6.92-6.90 (m, 1H), 6.74 (s, 1H), 6.66 (br d, 1H), 6.53 (s, 1H), 5.00-4.94 (m, 1H), 4.91-4.86 (m, 1H), 4.85 (br d, 1H), 4.70 (br d, 1H), 4.50 (br t, 1H), 4.48-4.44 (m, 1H), 4.41-4.35 (m, 1H), 4.12-4.08 (m, 1H), 4.08-4.04 (m, 1H), 3.78-3.70 (m, 1H), 3.69-3.63 (m, 1H), 3.57-3.56 (m, 1H), 3.61-3.54 (m, 3H), 3.51 (s, 3H), 3.51-3.48 (m, 3H), 3.48-3.44 (m, 2H), 3.43 (br t, 2H), 3.25-3.17 (m, 2H), 3.10 (s, 3H), 2.93 (q, 2H), 2.69-2.63 (m, 1H), 2.60 (br dd, 2H), 2.54 (s, 16H), 2.53-2.52 (m, 1H), 2.40-2.26 (m, 4H), 2.11 (br dd, 1H), 1.99-1.93 (m, 2H), 1.93-1.83 (m, 2H), 1.75-1.66 (m, 1H), 1.43 (d, 3H), 1.37 (sxt, 2H), 0.85 (d, 3H), 0.84-0.83 (m, 3H), 0.83-0.80 (m, 4H).

Synthesis of Compound 4

The synthesis of Compound 4 is according to the scheme depicted below.

This is a provisional file, not the final typeset article

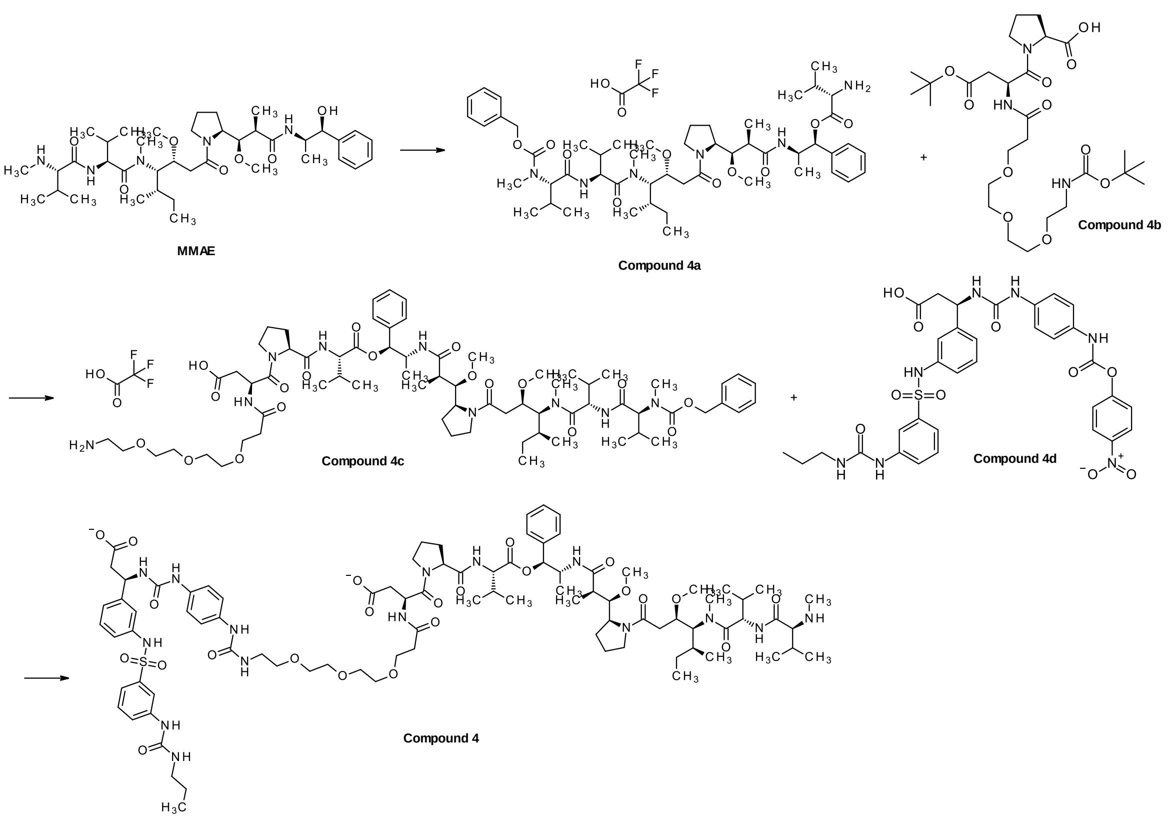

a) Synthesis of Compound 4a

Trifluoroacetic acid.N-[(benzyloxy)carbonyl]-N-methyl-L-valyl-N-[(3R,4S,5S)-3-methoxy-1-{(2S)-2-[(1R,2R)-1-methoxy-2-methyl-3-oxo-3-{[(1S,2R)-1-phenyl-1-(L-valyloxy)propan-2-yl]amino}propyl]pyrrolidin-1-yl}-5-methyl-1-oxoheptan-4-yl]-N-methyl-L-valinamide (1/1)

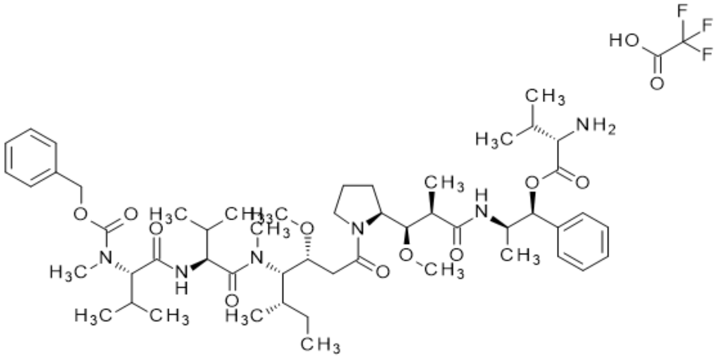

Step 1: To a solution of **Monomethyl auristatin E** (N-methyl-L-valyl-N-[(3R,4S,5S)-1-{(2S)-2-[(1R,2R)-3-{[(1S,2R)-1-hydroxy-1-phenylpropan-2-yl]amino}-1-methoxy-2-methyl-3-oxopropyl]pyrrolidin-1-yl}-3-methoxy-5-methyl-1-oxoheptan-4-yl]-N-methyl-L-valinamide, 200 mg, 279 µmol) in DMF (28 ml) were added 1-{[(benzyloxy)carbonyl]oxy}pyrrolidine-2,5-dione (83.3 mg, 334 µmol) and DIEA (150 µl, 840 µmol). The mixture was stirred at rt for 20 h and then concentrated under reduced pressure. The residue was purified by preparative HPLC and lyophilized to afford N-

[(benzyloxy)carbonyl]-N-methyl-L-valyl-N-[(3R,4S,5S)-1-{(2S)-2-[(1R,2R)-3-{[(1S,2R)-1-hydroxy-1-phenylpropan-2-yl]amino}-1-methoxy-2-methyl-3-oxopropyl]pyrrolidin-1-yl}-3-methoxy-5-methyl-1-oxoheptan-4-yl]-N-methyl-L-valinamide (224 mg, 100 % purity, 94 % yield). LC-MS (Method 1): R_t = 2.29 min; MS (ESIpos): m/z = 853 $[M+H]^+$.

Step 2: To a solution of N-[(benzyloxy)carbonyl]-N-methyl-L-valyl-N-[(3R,4S,5S)-1-{(2S)-2-[(1R,2R)-3-{[(1S,2R)-1-hydroxy-1-phenylpropan-2-yl]amino}-1-methoxy-2-methyl-3-oxopropyl]pyrrolidin-1-yl}-3-methoxy-5-methyl-1-oxoheptan-4-yl]-N-methyl-L-valinamide (222 mg, 100 % purity, 260 µmol) in DCM (40 ml) were added tert-butyl (4S)-4-methyl-2,5-dioxo-1,3-oxazolidine-3-carboxylate (190 mg, 781 µmol) and DMAP (63.6 mg, 521 µmol). The mixture was refluxed for 16 h and then concentrated under reduced pressure. The residue was purified by preparative HPLC and lyophilized to afford N-[(benzyloxy)carbonyl]-N-methyl-L-valyl-N-[(3R,4S,5S)-1-{(2S)-2-[(1R,2R)-3-{[(1S,2R)-1-{[N-(tert-butoxycarbonyl)-L-valyl]oxy}-1-phenylpropan-2-yl]amino}-1-methoxy-2-methyl-3-oxopropyl]pyrrolidin-1-yl}-3-methoxy-5-methyl-1-oxoheptan-4-yl]-N-methyl-L-valinamide (267 mg, 100 % purity, 98 % yield). LC-MS (Method 2): R_t = 6.35 min; MS (ESIpos): m/z = 1052 $[M+H]^+$.

Step 3: To a solution of N-[(benzyloxy)carbonyl]-N-methyl-L-valyl-N-[(3R,4S,5S)-1-{(2S)-2-[(1R,2R)-3-{[(1S,2R)-1-{[N-(tert-butoxycarbonyl)-L-valyl]oxy}-1-phenylpropan-2-yl]amino}-1-methoxy-2-methyl-3-oxopropyl]pyrrolidin-1-yl}-3-methoxy-5-methyl-1-oxoheptan-4-yl]-N-methyl-L-valinamide (323 mg, 100 % purity, 307 µmol) in DCM (50 ml), was added TFA (5.0 ml). The mixture was stirred at rt for 2 h and then concentrated under reduced pressure. The residue was dissolved in ACN/H_2O and lyophilized to afford **Compound 4a** (339 mg, 100 % purity, quant.) as an amorphous residue. LC-MS (Method 1): R_t = 1.80 min; MS (ESIpos): m/z = 952 $[M+H]^+$.

b) *Synthesis of Compound 4b*

(2S)-1-[(19S)-19-(2-tert-butoxy-2-oxoethyl)-2,2-dimethyl-4,17,20-trioxo-3,8,11,14-tetraoxa-5,18-diazaicosan-20-yl]pyrrolidine-2-carboxylic acid

This is a provisional file, not the final typeset article

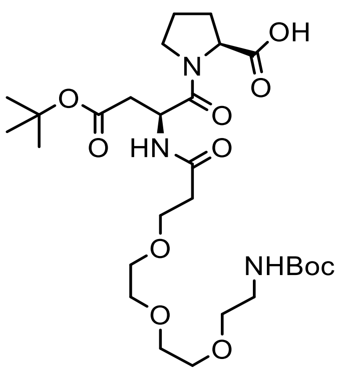

Compound 4b was synthesized using classical methods of peptide synthesis starting with the coupling of Z-Asp(OtBu)-OH with benzyl L-prolinate hydrochloride (1:1) in THF in the presence of T3P and DIPEA and subsequent removal of the Z-protecting group as well as the benzyl ester by hydrogenolysis over Pd/C to give (2S)-1-[(2S)-2-amino-4-tert-butoxy-4-oxobutanoyl]pyrrolidine-2-carboxylic acid. This partially protected dipeptide was acylated with tert-butyl{2-[2-(2-{3-[(2,5-dioxopyrrolidin-1-yl)oxy]-3-oxopropoxy}ethoxy)ethoxy]ethyl} carbamate to give the title compound. Tert-butyl{2-[2-(2-{3-[(2,5-dioxopyrrolidin-1-yl)oxy]-3-oxopropoxy}ethoxy) ethoxy]ethyl}carbamate was previously prepared by reacting 2,2-dimethyl-4-oxo-3,8,11,14-tetraoxa-5-azaheptadecan-17-oic acid with N-Hydroxysuccinimide in dioxane in the presence of EDCI. LC-MS: R_t = 0.81 min; MS (ESIpos): m/z = 590 $[M+H]^+$.

c) Synthesis of Compound 4c

(1S,2R)-2-({(2R,3R)-3-[(2S)-1-{(3R,4S,5S)-4-[{N-[(benzyloxy)carbonyl]-N-methyl-L-valyl-L-valyl}(methyl)amino]-3-methoxy-5-methylheptanoyl}pyrrolidin-2-yl]-3-methoxy-2-methylpropanoyl}amino)-1-phenylpropyl N-(3-{2-[2-(2-aminoethoxy)ethoxy]ethoxy}propanoyl)-L-alpha-aspartyl-L-prolyl-L-valinate.trifluoroacetic acid (1/1)

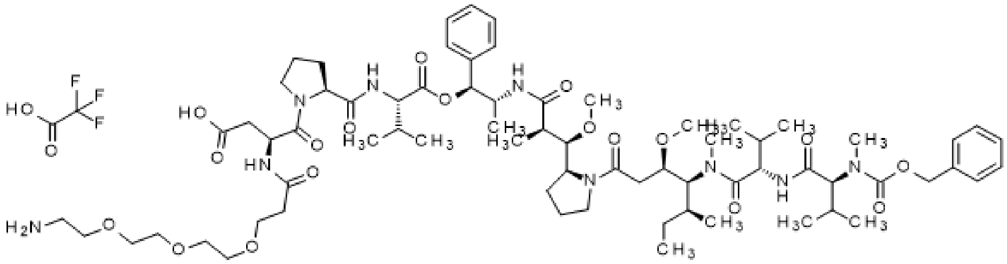

Step 1: To a solution of trifluoroacetic acid.N-[(benzyloxy)carbonyl]-N-methyl-L-valyl-N-[(3R,4S,5S)-3-methoxy-1-{(2S)-2-[(1R,2R)-1-methoxy-2-methyl-3-oxo-3-{[(1S,2R)-1-phenyl-1-(L-valyloxy)propan-2-yl]amino}propyl]pyrrolidin-1-yl}-5-methyl-1-oxoheptan-4-yl]-N-methyl-L-valinamide (1/1) (339 mg, 100 % purity, 318 µmol) (**Compound 4a**) in DMF (20 ml) were added (2S)-1-[(19S)-19-(2-tert-butoxy-2-oxoethyl)-2,2-dimethyl-4,17,20-trioxo-3,8,11,14-tetraoxa-5,18-

diazaicosan-20-yl]pyrrolidine-2-carboxylic acid (206 mg, 350 µmol) (**Compound 4b**), HATU (193 mg, 508 µmol) and DIEA (170 µl, 950 µmol). The mixture was stirred at rt for 1h30 and then concentrated under reduced pressure. The residue was purified by preparative HPLC and lyophilized to afford tert-butyl (19S)-19-[(2S)-2-({(2S)-1-[(1S,2R)-2-{[(2R,3R)-3-{(2S)-1-[(5S,8S,11S,12R)-11-[(2S)-butan-2-yl]-12-methoxy-4,10-dimethyl-3,6,9,14-tetraoxo-1-phenyl-5,8-di(propan-2-yl)-2-oxa-4,7,10-triazatetradecan-14-yl]pyrrolidin-2-yl}-3-methoxy-2-methylpropanoyl]amino}-1-phenylpropoxy]-3-methyl-1-oxobutan-2-yl}carbamoyl)pyrrolidine-1-carbonyl]-2,2-dimethyl-4,17-dioxo-3,8,11,14-tetraoxa-5,18-diazahenicosan-21-oate (447 mg, 100 % purity, 92 % yield) as an amorphous residue. LC-MS (Method 2): R_t = 6.26 min; MS (ESIpos): m/z = 1523 $[M+H]^+$.

Step 2: To a solution of tert-butyl (19S)-19-[(2S)-2-({(2S)-1-[(1S,2R)-2-{[(2R,3R)-3-{(2S)-1-[(5S,8S,11S,12R)-11-[(2S)-butan-2-yl]-12-methoxy-4,10-dimethyl-3,6,9,14-tetraoxo-1-phenyl-5,8-di(propan-2-yl)-2-oxa-4,7,10-triazatetradecan-14-yl]pyrrolidin-2-yl}-3-methoxy-2-methylpropanoyl]amino}-1-phenylpropoxy]-3-methyl-1-oxobutan-2-yl}carbamoyl)pyrrolidine-1-carbonyl]-2,2-dimethyl-4,17-dioxo-3,8,11,14-tetraoxa-5,18-diazahenicosan-21-oate (447 mg, 100 % purity, 294 µmol) in DCM (50 ml), was added TFA (5 ml). The mixture was stirred at rt for 2 h and then concentrated under reduced pressure. The residue was dissolved in ACN/H_2O and lyophilized to afford **Compound 4c** (497 mg, 93 % purity, quant.) as an amorphous residue. LC-MS (Method 2): R_t = 4.16 min; MS (ESIpos): m/z = 1367 $[M+H]^+$.

d) Synthesis of Compound 4

Disodium (1S,2R)-2-({(2R,3R)-3-methoxy-3-[(2S)-1-{(3R,4S,5S)-3-methoxy-5-methyl-4-[methyl(N-methyl-L-valyl-L-valyl)amino]heptanoyl}pyrrolidin-2-yl]-2-methylpropanoyl}amino)-1-phenylpropyl 1-[(2S)-2-(carboxylatomethyl)-17-{4-[({(1R)-2-carboxylato-1-[3-({3-[(propylcarbamoyl)amino]benzene-1-sulfonyl}amino)phenyl]ethyl}carbamoyl)amino]anilino}-4,17-dioxo-7,10,13-trioxa-3,16-diazaheptadecanan-1-oyl]-L-prolyl-L-valinate

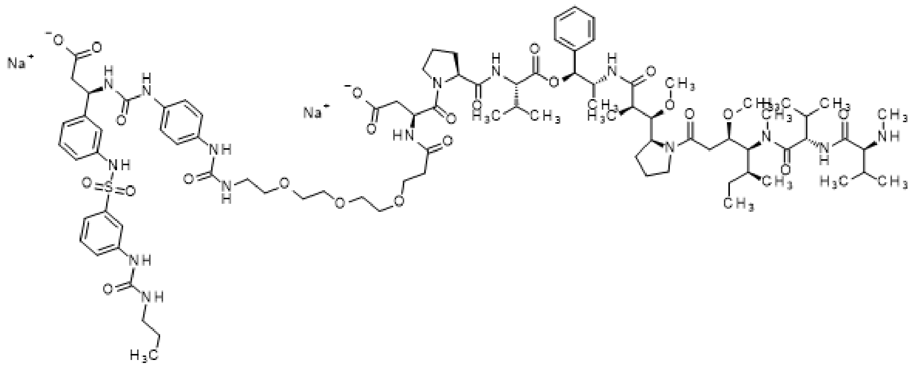

Step 1: To a solution of (1S,2R)-2-({(2R,3R)-3-[(2S)-1-{(3R,4S,5S)-4-[{N-[(benzyloxy)carbonyl]-N-methyl-L-valyl-L-valyl}(methyl)amino]-3-methoxy-5-methylheptanoyl}pyrrolidin-2-yl]-3-methoxy-2-methylpropanoyl}amino)-1-phenylpropyl N-(3-{2-[2-(2-aminoethoxy)ethoxy]ethoxy}propanoyl)-L-alpha-aspartyl-L-prolyl-L-valinate.trifluoroacetic acid (1/1) (246 mg, 93 % purity, 154 µmol) (**Compound 4c**) in DMF (40 ml), were added (3R)-3-{[(4-{[(4-nitrophenoxy)carbonyl]amino}phenyl)carbamoyl]amino}-3-[3-({3-[(propylcarbamoyl)amino]benzene-1-sulfonyl}amino)phenyl]propanoic acid (175 mg, 95 % purity, 231 µmol) (**Compound 4d**) and DIEA (130 µl, 770 µmol). The mixture was stirred at rt for 1h and then concentrated under reduced pressure. The synthesis of **Compound 4d** has been described in WO2020/094471. The residue was purified over preparative HPLC and freeze dried to afford (1S,2R)-2-({(2R,3R)-3-[(2S)-1-{(3R,4S,5S)-4-[{N-[(benzyloxy)carbonyl]-N-methyl-L-valyl-L-valyl}(methyl)amino]-3-methoxy-5-methylheptanoyl}pyrrolidin-2-yl]-3-methoxy-2-methylpropanoyl}amino)-1-phenylpropyl N-(14-{4-[({(1R)-2-carboxy-1-[3-({3-[(propylcarbamoyl)amino]benzene-1-sulfonyl}amino)phenyl]ethyl}carbamoyl)amino]anilino}-14-oxo-4,7,10-trioxa-13-azatetradecanan-1-oyl)-L-alpha-aspartyl-L-prolyl-L-valinate (241 mg, 97 % purity, 78 % yield) as an amorphous residue. LC-MS (Method 2): R_t = 5.15 min; MS (ESIpos): m/z = 1947 $[M+H]^+$.

Step 2: (1S,2R)-2-({(2R,3R)-3-[(2S)-1-{(3R,4S,5S)-4-[{N-[(benzyloxy)carbonyl]-N-methyl-L-valyl-L-valyl}(methyl)amino]-3-methoxy-5-methylheptanoyl}pyrrolidin-2-yl]-3-methoxy-2-methylpropanoyl}amino)-1-phenylpropyl N-(14-{4-[({(1R)-2-carboxy-1-[3-({3-[(propylcarbamoyl)amino]benzene-1-sulfonyl}amino)phenyl]ethyl}carbamoyl)amino]anilino}-14-oxo-4,7,10-trioxa-13-azatetradecanan-1-oyl)-L-alpha-aspartyl-L-prolyl-L-valinate (189 mg, 97.3 µmol) was dissolved in ethanol (30 ml). Pd/C 10% (18.9 mg) was added, and the reaction was hydrogenated at RT for 4h30 and filtered. The mother liquor was concentrated *in vacuo,* purified over preparative HPLC and lyophilized to give (1S,2R)-2-({(2R,3R)-3-methoxy-3-[(2S)-1-{(3R,4S,5S)-3-methoxy-5-methyl-4-[methyl(N-methyl-L-valyl-L-valyl)amino]heptanoyl}pyrrolidin-2-yl]-2-methylpropanoyl}amino)-1-phenylpropyl N-(14-{4-[({(1R)-2-carboxy-1-[3-({3-[(propylcarbamoyl)amino]benzene-1-sulfonyl}amino)phenyl]ethyl}carbamoyl)amino]anilino}-14-oxo-4,7,10-trioxa-13-azatetradecanan-1-oyl)-L-alpha-aspartyl-L-prolyl-L-valinate (116 mg, 94 % purity, 61 % yield) as an amorphous residue. LC-MS (Method 2): R_t = 3.49 min; MS (ESIpos): m/z = 1813 $[M+H]^+$.

Step 3: To a solution of (1S,2R)-2-({(2R,3R)-3-methoxy-3-[(2S)-1-{(3R,4S,5S)-3-methoxy-5-methyl-4-[methyl(N-methyl-L-valyl-L-valyl)amino]heptanoyl}pyrrolidin-2-yl]-2-methylpropanoyl}amino)-1-phenylpropyl N-(14-{4-[({(1R)-2-carboxy-1-[3-({3-[(propylcarbamoyl)amino]benzene-1-sulfonyl}amino)phenyl]ethyl}carbamoyl)amino]anilino}-14-oxo-4,7,10-trioxa-13-azatetradecanan-1-oyl)-L-alpha-aspartyl-L-prolyl-L-valinate (25.0 mg, 94 % purity, 12.9 µmol) in dioxane / water (1:1, 14 mL) was added a sodium hydroxide solution (26 µl, 1 M, 26 µmol). The solution was freeze-dried to give **Compound 4** (23.9 mg, 96% purity, 96% yield) as a colorless foam.

LC-MS (Method 2): R_t = 3.49 min; MS (ESIpos): m/z = 1811 $[M+H]^+$.

^{1}H-NMR (400 MHz, DMSO-d_6) delta [ppm]: 11.28 (br s, 1H), 9.5-9.5 (m, 1H), 8.7-8.9 (m, 1H), 8.52 (s, 1H), 8.1-8.2 (m, 1H), 7.9-8.1 (m, 1H), 7.8-7.9 (m, 1H), 7.8-7.8 (m, 1H), 7.49 (br s, 1H), 7.3-7.3 (m, 1H), 7.28 (br t, 1H, J=4.3 Hz), 7.22 (br dd, 1H, J=1.9, 8.8 Hz), 7.1-7.2 (m, 1H), 7.0-7.1 (m, 1H), 6.9-7.0 (m, 1H), 6.63 (br dd, 1H, J=2.4, 3.9 Hz), 5.5-5.7 (m, 1H), 4.9-5.0 (m, 1H), 4.5-4.8 (m, 1H), 4.3-4.4 (m, 1H), 4.1-4.2 (m, 1H), 4.0-4.1 (m, 1H), 3.8-3.9 (m, 1H), 3.5-3.6 (m, 1H), 3.51 (br d, 2H, J=4.1 Hz), 3.4-3.5 (m, 4H), 3.3-3.3 (m, 1H), 3.2-3.3 (m, 1H), 3.2-3.2 (m, 1H), 3.1-3.2 (m, 1H), 3.0-3.0 (m, 1H), 2.92 (q, 1H, J=6.4 Hz), 2.5-2.6 (m, 8H), 2.1-2.2 (m, 1H), 1.7-2.0 (m, 2H), 1.5-1.6 (m, 1H), 1.3-1.4 (m, 1H), 1.0-1.1 (m, 2H), 0.9-0.9 (m, 2H), 0.8-0.9 (m, 3H), 0.7-0.8 (m, 2H).

Synthesis of Compound 5

The synthesis of Compound 5 is according to the scheme depicted below.

This is a provisional file, not the final typeset article

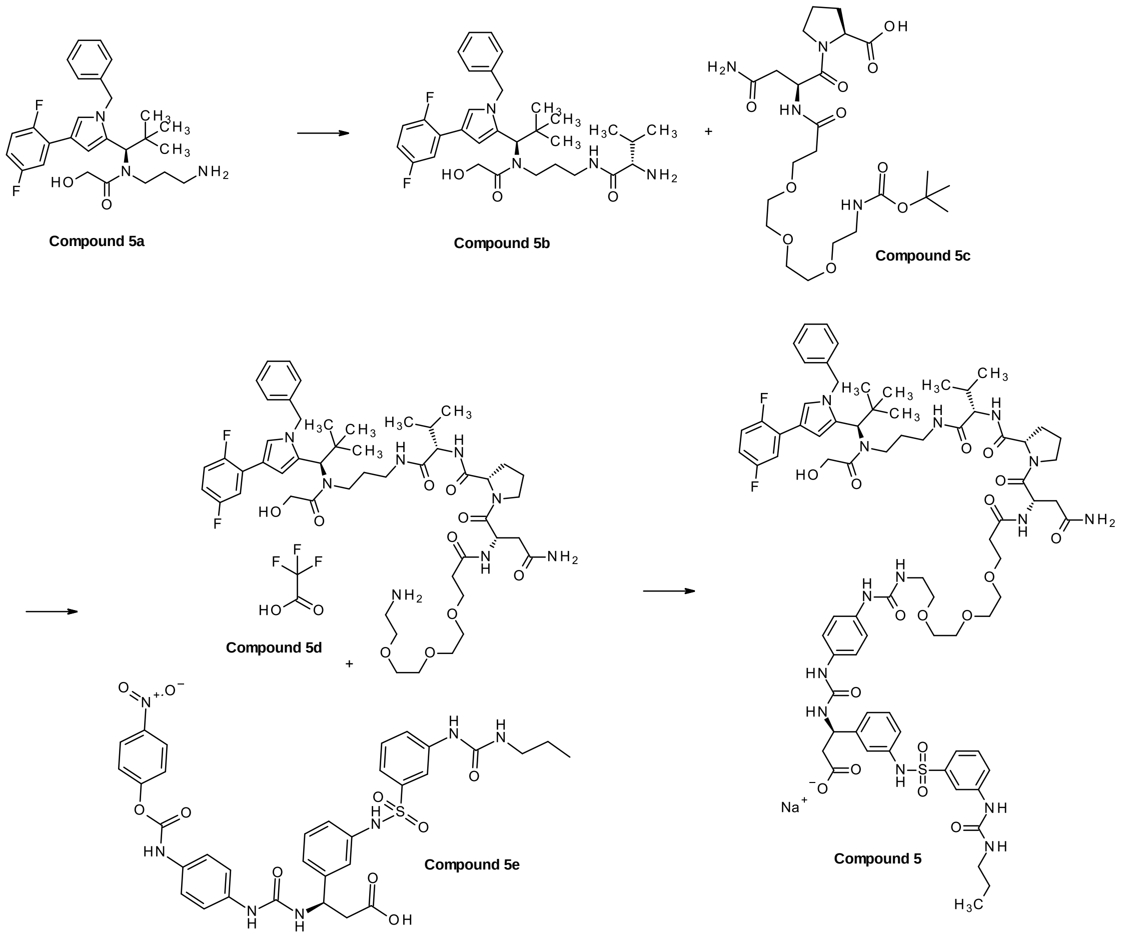

a) Synthesis of Compound 5b

N-{3-[{(1R)-1-[1-benzyl-4-(2,5-difluorophenyl)-1H-pyrrol-2-yl]-2,2-dimethylpropyl}(hydroxyacetyl)amino]propyl}-L-valinamide

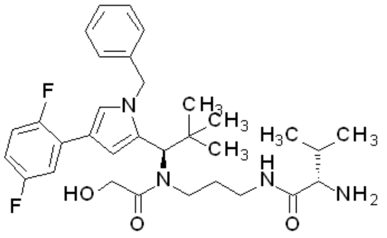

Step 1: To a solution of trifluoroacetic acid.N-(3-aminopropyl)-N-{(1R)-1-[1-benzyl-4-(2,5-difluorophenyl)-1H-pyrrol-2-yl]-2,2-dimethylpropyl}-2-hydroxyacetamide (1/1) (250 mg, 428 µmol, as described in WO2015096982) in DMF (20 ml) were added 2,5-dioxopyrrolidin-1-yl N-[(benzyloxy)carbonyl]-L-valinate (298 mg, 857 µmol) and DIEA (300 µl, 1.7 mmol). The mixture was stirred at rt for 1 h and then diluted with water and ethyl acetate. The layers were separated, and the organic phase was then concentrated under reduced pressure. The residue was purified over preparative

HPLC and lyophilized to N-{3-[{(1R)-1-[1-benzyl-4-(2,5-difluorophenyl)-1H-pyrrol-2-yl]-2,2-dimethylpropyl}(hydroxyacetyl)amino]propyl}-N^2-[(benzyloxy)carbonyl]-L-valinamide (252 mg, 98% purity, 82% yield) as an amorphous residue. LC-MS (Method 2): R_t = 5.72 min; MS (ESIpos): m/z = 703 $[M+H]^+$.

Step 2: To a solution of N-{3-[{(1R)-1-[1-benzyl-4-(2,5-difluorophenyl)-1H-pyrrol-2-yl]-2,2-dimethylpropyl}(hydroxyacetyl)amino]propyl}-N^2-[(benzyloxy)carbonyl]-L-valinamide (250 mg, 98 % purity, 349 µmol) in DCM (15 ml) and methanol (15 ml) was added Pd/C (45 mg). The reaction was hydrogenated at rt for 2h30 and filtered over celite. The mother liquor was concentrated *in vacuo* to give **Compound 5b** (117 mg, 100% purity, 59% yield) as a colorless resin which was used in the next step without further purification.

b) Synthesis of Compound 5c

(2S)-1-[(2S)-4-amino-2-[3-[2-[2-[2-(tert-butoxycarbonylamino)ethoxy]ethoxy]ethoxy]propanoylamino]-4-oxo-butanoyl]pyrrolidine-2-carboxylic acid

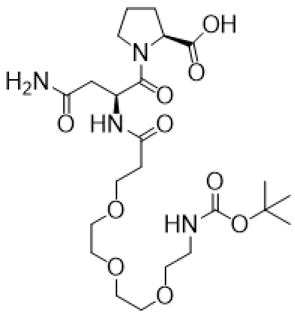

Compound 5c was synthesized using classical peptide synthesis methods starting with the coupling of (2S)-4-amino-2-(tert-butoxycarbonylamino)-4-oxo-butanoic acid with benzyl (2S)-pyrrolidine-2-carboxylate.hydrochloride in DMF in the presence of N,N-Diisopropylethylamine and subsequent removal of the Boc-protecting group with TFA in DCM. This partially protected dipeptide was acylated with tert-butyl {2-[2-(2-{3-[(2,5-dioxopyrrolidin-1-yl)oxy]-3-oxopropoxy}ethoxy)ethoxy]ethyl}carbamate in DMF in the presence of N, N-diisopropylethylamine.

For the synthesis of tert-butyl {2-[2-(2-{3-[(2,5-dioxopyrrolidin-1-yl)oxy]-3-oxopropoxy}ethoxy)ethoxy]ethyl}carbamate, to a suspension of 2,2-dimethyl-4-oxo-3,8,11,14-tetraoxa-5-azaheptadecan-17-oic acid (3.00 g, 9.33 mmol) in DCM (50 ml), were added 1-hydroxypyrrolidine-2,5-dione (1.61 g, 14.0 mmol), EDCI (2.15 g, 11.2 mmol) and DMAP (5.00 mg, 40.9 µmol). The reaction was stirred for 1 hour at RT and concentrated *in vacuo*. The residue was purified by preparative HPLC to give tert-butyl {2-[2-(2-{3-[(2,5-dioxopyrrolidin-1-yl)oxy]-3-

This is a provisional file, not the final typeset article

oxopropoxy}ethoxy)ethoxy]ethyl}carbamate (3.15 g, 90 % purity, 72 % yield) as a colorless oil. LC-MS (Method 1): R_t = 1.38 min; MS (ESIpos): m/z = 419 $[M+H]^+$.

In the final step of the synthesis of **Compound 5c** the benzylester was removed by hydrogenolysis over 10% Pd/charcoal. LC-MS (Method 1): R_t = 1.00 min; MS (ESIpos): m/z = 533 $[M+H]^+$.

c) Synthesis of Compound 5d

Trifluoroacetic acid.N^2-(3-{2-[2-(2-aminoethoxy)ethoxy]ethoxy}propanoyl)-L-asparaginyl-L-prolyl-N-{3-[{(1R)-1-[1-benzyl-4-(2,5-difluorophenyl)-1H-pyrrol-2-yl]-2,2-dimethylpropyl}(hydroxyacetyl)amino]propyl}-L-valinamide (1/1)

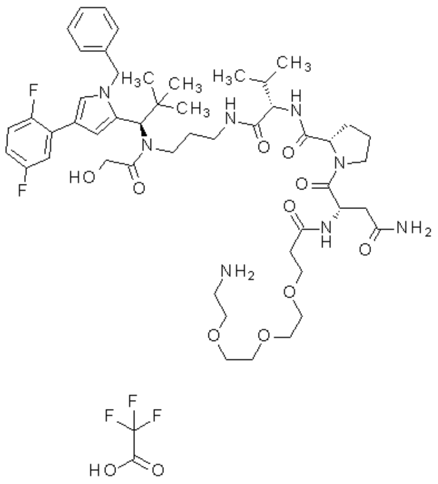

Step 1: To a solution of N-{3-[{(1R)-1-[1-benzyl-4-(2,5-difluorophenyl)-1H-pyrrol-2-yl]-2,2-dimethylpropyl}(hydroxyacetyl)amino]propyl}-L-valinamide (115 mg, 202 µmol) (**Compound 5b**) in DMF (20 ml) were added N^2-(2,2-dimethyl-4,17-dioxo-3,8,11,14-tetraoxa-5-azaheptadecan-17-yl)-L-asparaginyl-L-proline (140 mg, 92 % purity, 243 µmol) (**Compound 5c**), HATU (100 mg, 263 µmol) and DIEA (110 µl, 610 µmol). The mixture was stirred at rt for 2h and then concentrated under reduced pressure. The residue was purified by preparative HPLC and lyophilized to afford N^2-(2,2-dimethyl-4,17-dioxo-3,8,11,14-tetraoxa-5-azaheptadecan-17-yl)-L-asparaginyl-L-prolyl-N-{3-[{(1R)-1-[1-benzyl-4-(2,5-difluorophenyl)-1H-pyrrol-2-yl]-2,2-dimethylpropyl}(hydroxyacetyl)amino]propyl}-L-valinamide (153 mg, 76 % purity, 53 % yield) as an amorphous residue. LC-MS (Method 2): R_t = 5.01 min; MS (ESIpos): m/z = 1084 $[M+H]^+$.

Step 2: To a solution of N^2-(2,2-dimethyl-4,17-dioxo-3,8,11,14-tetraoxa-5-azaheptadecan-17-yl)-L-asparaginyl-L-prolyl-N-{3-[{(1R)-1-[1-benzyl-4-(2,5-difluorophenyl)-1H-pyrrol-2-yl]-2,2-dimethylpropyl}(hydroxyacetyl)amino]propyl}-L-valinamide (280 mg, 96 % purity, 247 µmol) in Trifluoroethanol (20 ml), was added zinc chloride (269 mg, 1.98 mmol). The mixture was stirred at 50°C for 5 h. EDTA (577 mg, 1.98 mmol) and water 0.1% TFA (6 ml) were then added, and the

resulting mixture was purified over preparative HPLC and lyophilized to afford **Compound 5d** (192 mg, 100 % purity, 71% yield) as an amorphous residue. LC-MS (Method 2): R_t = 3.62 min; MS (ESIpos): m/z = 984 $[M+H]^+$.

d) Synthesis of Compound 5

Sodium N^2-(14-{4-[({(1R)-2-carboxylato-1-[3-({3-[(propylcarbamoyl)amino]benzene-1-sulfonyl}amino)phenyl]ethyl}carbamoyl)amino]anilino}-14-oxo-4,7,10-trioxa-13-azatetradecanan-1-oyl)-L-asparaginyl-L-prolyl-N-{3-[{(1R)-1-[1-benzyl-4-(2,5-difluorophenyl)-1H-pyrrol-2-yl]-2,2-dimethylpropyl}(hydroxyacetyl)amino]propyl}-L-valinamide

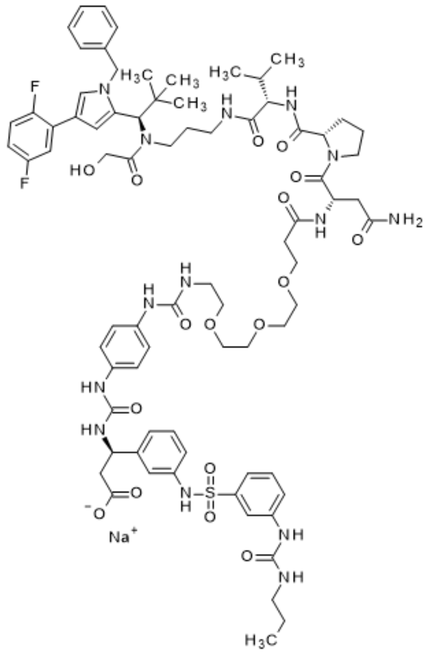

To a solution of trifluoroacetic acid-N2-(3-{2-[2-(2-aminoethoxy)ethoxy]ethoxy}propanoyl)-L-asparaginyl-L-prolyl-N-{3-[{(1R)-1-[1-benzyl-4-(2,5-difluorophenyl)-1H-pyrrol-2-yl]-2,2-dimethylpropyl}(hydroxyacetyl)amino]propyl}-L-valinamide (1/1) (15.0 mg, 13.7 µmol) (**Compound 5d**) in DMF (6.8 ml), were added (3R)-3-{[(4-{[(4-nitrophenoxy)carbonyl]amino}phenyl)carbamoyl]amino}-3-[3-({3-[(propylcarbamoyl)amino]benzene-1-sulfonyl}amino)phenyl]propanoic acid (9.84 mg, 13.7 µmol) (**Compound 5e**) and DIEA (24 µl, 140 µmol). The synthesis of **Compound 5e** has been described in WO2020/094471. The mixture was stirred at rt for 30 min and then concentrated under reduced pressure. The residue was purified over preparative HPLC and freeze dried to afford an intermediate compound (12 mg, 100% purity, 56% yield) as a colorless foam LC-MS (Method 2): R_t = 4.74 min; MS (ESIpos): m/z = 1563 $[M+H]^+$.

This is a provisional file, not the final typeset article

To a solution of this intermediate (100 mg, 93 % purity, 59.6 µmol) in dioxane / water (1:1, 20 mL) was added a sodium hydroxide solution (600 µl, 0.1 M, 60 µmol). The solution was freeze-dried to give **Compound 5** (100 mg, 95% purity, quant.) as a colorless foam.

LC-MS (Method 2): R_t = 4.72 min; MS (ESIpos): m/z = 1563 $[M+H]^+$.

^{1}H-NMR (600 MHz, DMSO-d6) delta [ppm]: 12.2-12.4 (m, 1H), 10.19 (br s, 1H), 8.4-8.5 (m, 1H), 8.33 (s, 1H), 8.17 (br d, 1H, J=7.9 Hz), 7.95 (br d, 1H, J=1.7 Hz), 7.67 (br s, 1H), 7.6-7.7 (m, 1H), 7.6-7.6 (m, 1H), 7.5-7.6 (m, 2H), 7.50 (br s, 1H), 7.41 (br s, 1H), 7.3-7.4 (m, 2H), 7.3-7.3 (m, 2H), 7.27 (br d, 1H, J=2.4 Hz), 7.24 (br s, 1H), 7.2-7.2 (m, 1H), 7.2-7.2 (m, 6H), 7.1-7.2 (m, 1H), 6.98 (br d, 2H, J=8.2 Hz), 6.9-7.0 (m, 2H), 6.85 (br d, 1H, J=7.9 Hz), 6.7-6.8 (m, 1H), 6.08 (br t, 1H, J=5.2 Hz), 5.60 (s, 1H), 5.17 (br d, 1H, J=16.0 Hz), 5.0-5.0 (m, 1H), 4.90 (br d, 1H, J=15.7 Hz), 4.80 (q, 1H, J=7.3 Hz), 4.59 (t, 1H, J=5.6 Hz), 4.3-4.4 (m, 1H), 4.27 (br dd, 1H, J=5.3, 15.7 Hz), 4.02 (br dd, 1H, J=5.7, 15.7 Hz), 3.90 (t, 1H, J=7.9 Hz), 3.6-3.7 (m, 2H), 3.57 (br t, 2H, J=6.6 Hz), 3.52 (s, 4H), 3.48 (br dd, 4H, J=2.2, 6.7 Hz), 3.43 (br t, 2H, J=5.7 Hz), 3.2-3.3 (m, 2H), 3.01 (q, 2H, J=6.5 Hz), 2.6-2.8 (m, 2H), 2.6-2.6 (m, 2H), 2.3-2.4 (m, 3H), 1.9-2.0 (m, 1H), 1.8-1.9 (m, 4H), 1.42 (sxt, 2H, J=7.2 Hz), 1.1-1.3 (m, 1H), 0.8-0.9 (m, 4H), 0.82 (s, 8H), 0.78 (br d, 3H, J=6.8 Hz), 0.75 (br d, 3H, J=6.7 Hz), 0.3-0.4 (m, 1H).

Synthesis of Compound 6

The synthesis of Compound 6 is according to the scheme depicted below.

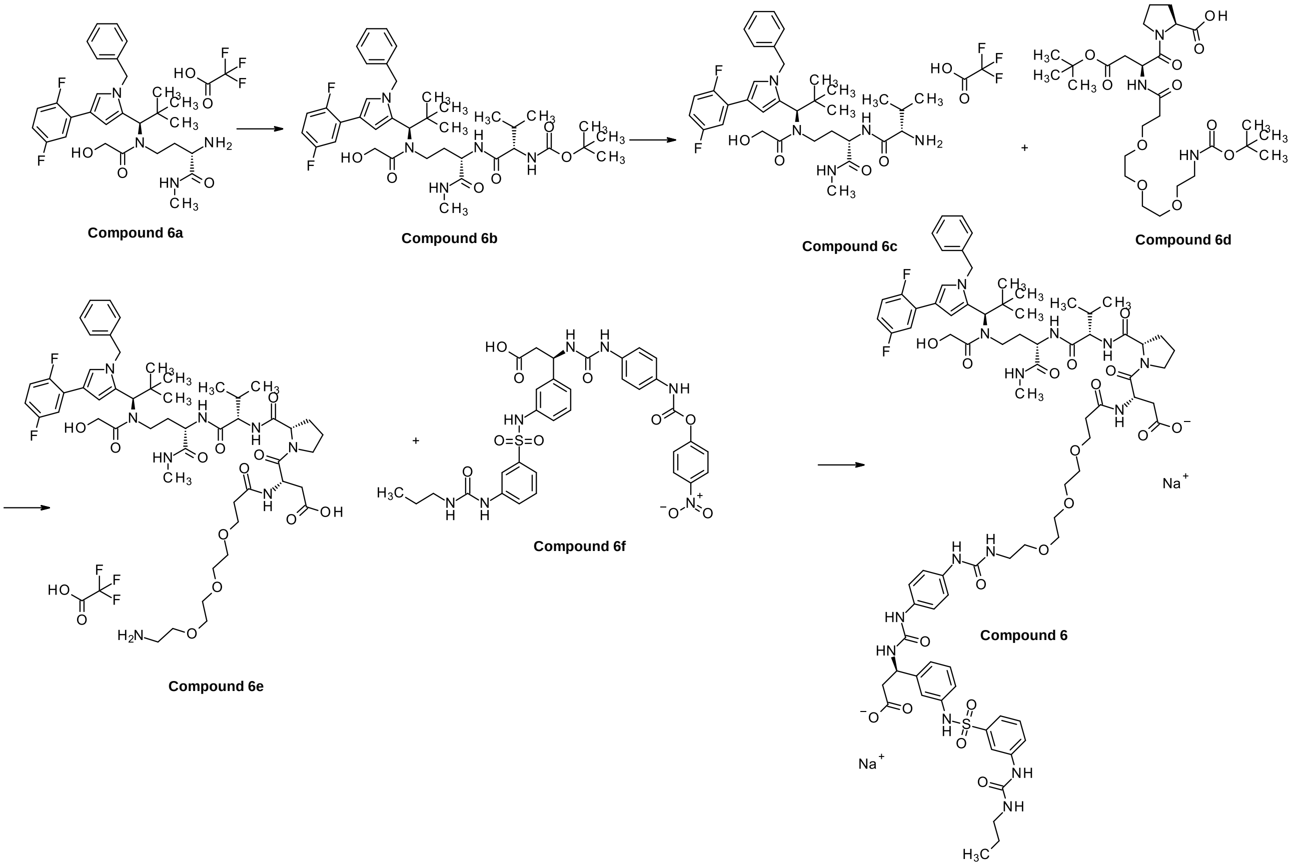

a) Synthesis of Compound 6a

(2S)-2-amino-4-[{(1R)-1-[1-benzyl-4-(2,5-difluorophenyl)-1H-pyrrol-2-yl]-2,2-dimethylpropyl}(glycoloyl)amino]-N-methylbutanamide trifluoroacetate (1:1)

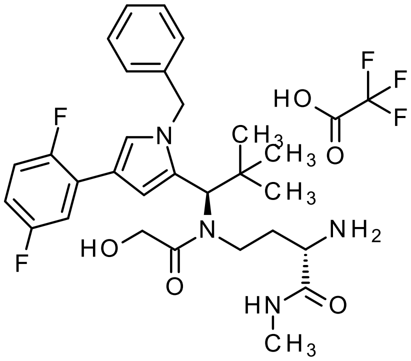

The synthesis of Compound 6a has been described in WO2015/096982.

b) Synthesis of Compound 6b

N-[(2S)-4-[{(1R)-1-[1-benzyl-4-(2,5-difluorophenyl)-1H-pyrrol-2-yl]-2,2-dimethylpropyl}(glycoloyl)amino]-1-(methylamino)-1-oxobutan-2-yl]-N²-(tert-butoxycarbonyl)-L-valinamide

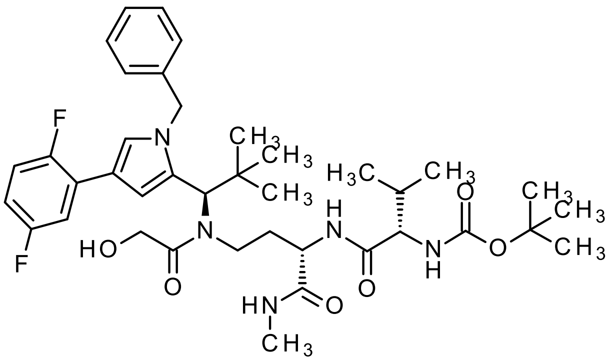

(2S)-2-amino-4-[{(1R)-1-[1-benzyl-4-(2,5-difluorophenyl)-1H-pyrrol-2-yl]-2,2-dimethylpropyl}(glycoloyl)amino]-N-methylbutanamide trifluoroacetate (**Compound 6a**) (200mg, 0.31mmol) was dissolved in DMF (5.0ml). Then N-(tert-butoxycarbonyl)valine (81.1mg, 0.373mmol), HATU (177.3mg, 0.466mmol) and DIEA (162.5uL, 0.933mmol) were added and the reaction was stirred at RT for 2h. The residue was purified via prep. HPLC and then lyophilized to give **Compound 6b** (210.0mg, 95% purity, 89% yield) as a white foam. LC-MS (Method 1): R_t = 2.47 min; MS (ESIpos): m/z = 724 $[M-H]^-$.

c) Synthesis of Compound 6c

N-[(2S)-4-[{(1R)-1-[1-benzyl-4-(2,5-difluorophenyl)-1H-pyrrol-2-yl]-2,2-dimethylpropyl}(glycoloyl)amino]-1-(methylamino)-1-oxobutan-2-yl]-L-valinamide trifluoroacetate (1:1)

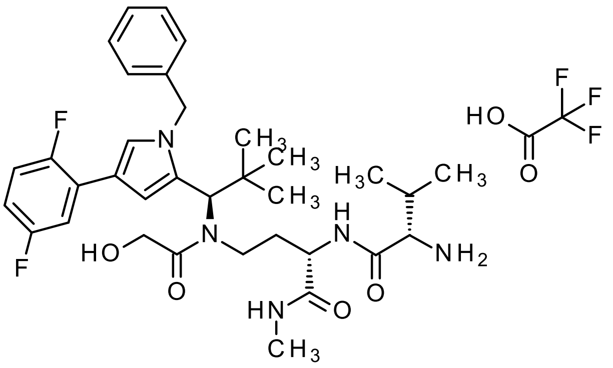

N-[(2S)-4-[{(1R)-1-[1-benzyl-4-(2,5-difluorophenyl)-1H-pyrrol-2-yl]-2,2-dimethylpropyl}(glycoloyl)amino]-1-(methylamino)-1-oxobutan-2-yl]-N²-(tert-

butoxycarbonyl)-L-valinamide (**Compound 6b**) (209.0mg, 0.275mmol) was dissolved in 2,2,2-trifluorethanol (10.0mL), $ZnCl_2$ (225.1mg, 1.65mmol) was added and the reaction was stirred for 2h at 50°C. EDTA (482.6mg, 1.65mmol) and 5ml H_2O + 0,1%TFA were added. The reaction was concentrated and purified via prep HPLC and then lyophilized to give **Compound 6c** (201.0mg, 100% purity, 98% yield) as a white foam. LC-MS (Method 3): R_t = 1.48 min; MS (ESIpos): m/z = 626 $[M+H]^+$.

d) Synthesis of Compound 6d

(2S)-1-[(19S)-19-(2-tert-butoxy-2-oxoethyl)-2,2-dimethyl-4,17,20-trioxo-3,8,11,14-tetraoxa-5,18-diazaicosan-20-yl]pyrrolidine-2-carboxylic acid

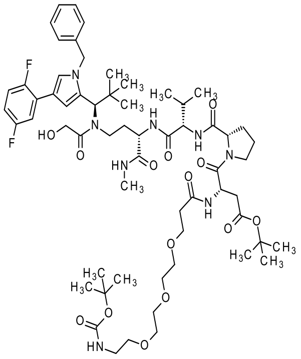

Compound 6d was synthesized using classical methods of peptide synthesis starting with the coupling of Z-Asp(OtBu)-OH with benzyl L-prolinate hydrochloride (1:1) in THF in the presence of T3P and DIPEA and subsequent removal of the Z-protecting group as well as the benzyl ester by hydrogenolysis over Pd/C to give (2S)-1-[(2S)-2-amino-4-tert-butoxy-4-oxobutanoyl] pyrrolidine-2-carboxylic acid. This partially protected dipeptide was acylated with tert-butyl{2-[2-(2-{3-[(2,5-dioxopyrrolidin-1-yl)oxy]-3-oxopropoxy}ethoxy)ethoxy]ethyl} carbamate to give the title compound. Tert-butyl{2-[2-(2-{3-[(2,5-dioxopyrrolidin-1-yl)oxy]-3-oxopropoxy}ethoxy) ethoxy]ethyl}carbamate was previously prepared by reacting 2,2-dimethyl-4-oxo-3,8,11,14-tetraoxa-5-azaheptadecan-17-oic acid with N-Hydroxysuccinimide in dioxane in the presence of EDCI. LC-MS: R_t = 0.81 min; MS (ESIpos): m/z = 590 $[M+H]^+$.

e) Synthesis of Compound 6e

This is a provisional file, not the final typeset article

N-(3-{2-[2-(2-aminoethoxy)ethoxy]ethoxy}propanoyl)-L-alpha-aspartyl-L-prolyl-N-[(2S)-4-[{(1R)-1-[1-benzyl-4-(2,5-difluorophenyl)-1H-pyrrol-2-yl]-2,2-dimethylpropyl}(glycoloyl)amino]-1-(methylamino)-1-oxobutan-2-yl]-L-valinamide trifluoroacetate (1:1)

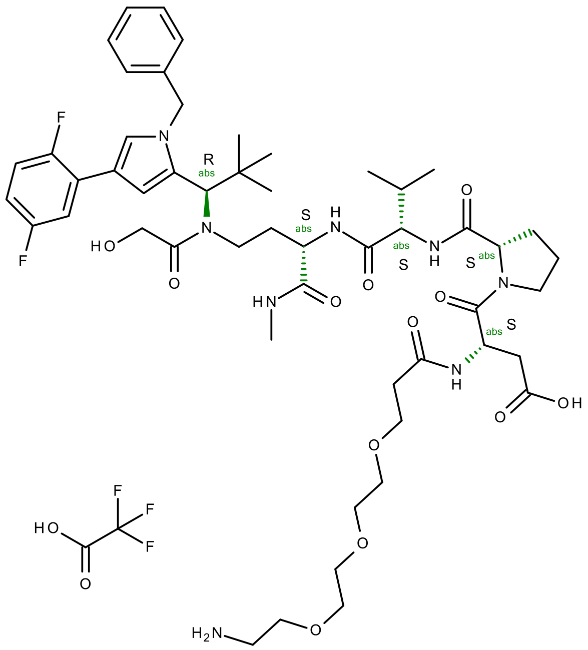

Step 1: N-[(2S)-4-[{(1R)-1-[1-benzyl-4-(2,5-difluorophenyl)-1H-pyrrol-2-yl]-2,2-dimethylpropyl}(glycoloyl)amino]-1-(methylamino)-1-oxobutan-2-yl]-L-valinamide trifluoroacetate (**Compound 6c**) (199.0mg, 0.269mmol) was dissolved in DMF (5.0ml). Then (2S)-1-[(19S)-19-(2-tert-butoxy-2-oxoethyl)-2,2-dimethyl-4,17,20-trioxo-3,8,11,14-tetraoxa-5,18-diazaicosan-20-yl]pyrrolidine-2-carboxylic acid (**Compound 6d**) (174.5mg, 0.296mmol), HATU (163.6mg, 0.43mmol) and DIEA (141uL, 0.807mmol) were added and the reaction was stirred at RT for overnight. The residue was purified via prep. HPLC and then lyophilized to give an intermediate compound (215.0mg, 99% purity, 66% yield) as a white foam. LC-MS (Method 1): R_t = 2.48 min; MS (ESIpos): m/z = 1195 $[M-H]^-$.

Step 2: This intermediate tert-butyl (19S)-19-{[(2S)-2-{[(2S)-1-{[(2S)-4-[{(1R)-1-[1-benzyl-4-(2,5-difluorophenyl)-1H-pyrrol-2-yl]-2,2-dimethylpropyl}(glycoloyl)amino]-1-(methylamino)-1-oxobutan-2-yl]amino}-3-methyl-1-oxobutan-2-yl]carbamoyl}pyrrolidin-1-yl]carbonyl}-2,2-dimethyl-4,17-dioxo-3,8,11,14-tetraoxa-5,18-diazahenicosan-21-oate (215.0 mg, 0.173 mmol) was then dissolved in 2,2,2-trifluorethanol (6.0ml), $ZnCl_2$ (141.7mg, 1.04mmol) was added and the reaction was stirred for 4h at 50°C. EDTA (303.8mg, 1.04 mmol) and ~6ml H2O+0,1%TFA were added. The solvent was reduced to 1/3 and the residue was purified via prep HPLC and then lyophilized to give

Compound 6e (182.0mg, 100% purity, 91% yield) as a white foam. LC-MS (Method 2): R_t = 3.64 min; MS (ESIpos): m/z = 1041 $[M+H]^+$.

f) Synthesis of Compound 6

Disodium 1-{(2S)-2-(carboxylatomethyl)-17-[4-({[(1R)-2-carboxylato-1-{3-[({3-[(propylcarbamoyl)amino]phenyl}sulfonyl)amino]phenyl}ethyl]carbamoyl}amino)anilino]-4,17-dioxo-7,10,13-trioxa-3,16-diazaheptadecan-1-oyl}-L-prolyl-N-[(2S)-4-[{(1R)-1-[1-benzyl-4-(2,5-difluorophenyl)-1H-pyrrol-2-yl]-2,2-dimethylpropyl}(glycoloyl)amino]-1-(methylamino)-1-oxobutan-2-yl]-L-valinamide

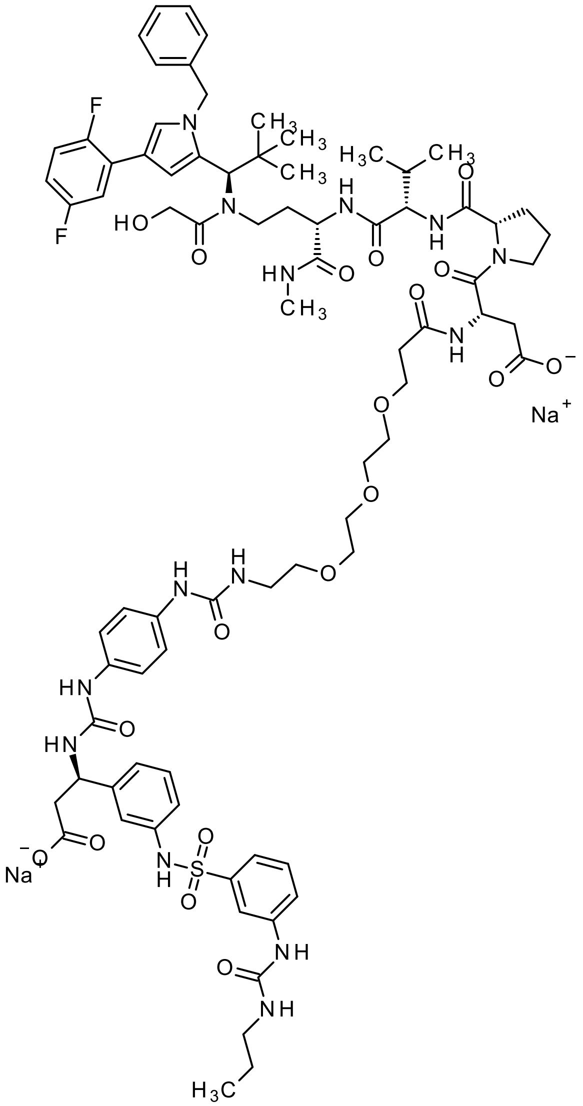

Step 1: To a solution of N-(3-{2-[2-(2-aminoethoxy)ethoxy]ethoxy}propanoyl)-L-alpha-aspartyl-L-prolyl-N-[(2S)-4-[{(1R)-1-[1-benzyl-4-(2,5-difluorophenyl)-1H-pyrrol-2-yl]-2,2-dimethylpropyl}(hydroxyacetyl)amino]-1-(methylamino)-1-oxobutan-2-yl]-L-

This is a provisional file, not the final typeset article

valinamide.trifluoroacetic acid (1/1) (20.0 mg, 100 % purity, 17.3 µmol) (**Compound 6e**) in DMF (2.1 ml), were added (3R)-3-{[(4-{[(4-nitrophenoxy)carbonyl]amino}phenyl)carbamoyl]amino}-3-[3-({3-[(propylcarbamoyl)amino]benzene-1-sulfonyl}amino)phenyl]propanoic acid (14.4 mg, 95 % purity, 19.0 µmol) (**Compound 6f**) and DIEA (30 µl, 170 µmol). The synthesis of **Compound 6f** has been described in WO2020/094471. The mixture was stirred at rt for 1h and then concentrated under reduced pressure. The residue was purified over preparative HPLC and freeze dried to afford N-(14-{4-[({(1R)-2-carboxy-1-[3-({3-[(propylcarbamoyl)amino]benzene-1-sulfonyl}amino)phenyl]ethyl}carbamoyl)amino]anilino}-14-oxo-4,7,10-trioxa-13-azatetradecanan-1-oyl)-L-alpha-aspartyl-L-prolyl-N-[(2S)-4-[{(1R)-1-[1-benzyl-4-(2,5-difluorophenyl)-1H-pyrrol-2-yl]-2,2-dimethylpropyl}(hydroxyacetyl)amino]-1-(methylamino)-1-oxobutan-2-yl]-L-valinamide (22.3 mg, 99 % purity, 78 % yield). LC-MS (Method 2): R_t = 4.72 min; MS (ESIpos): m/z = 1622 $[M+H]^+$.

Step 2: To a solution of N-(14-{4-[({(1R)-2-carboxy-1-[3-({3-[(propylcarbamoyl)amino]benzene-1-sulfonyl}amino)phenyl]ethyl}carbamoyl)amino]anilino}-14-oxo-4,7,10-trioxa-13-azatetradecanan-1-oyl)-L-alpha-aspartyl-L-prolyl-N-[(2S)-4-[{(1R)-1-[1-benzyl-4-(2,5-difluorophenyl)-1H-pyrrol-2-yl]-2,2-dimethylpropyl}(hydroxyacetyl)amino]-1-(methylamino)-1-oxobutan-2-yl]-L-valinamide (21.8 mg, 99 % purity, 13.2 µmol) in dioxane / water (1:1, 6 mL) was added a sodium hydroxide solution (26 µl, 1 M, 26 µmol). The solution was freeze-dried to give **Compound 6** (21.8 mg, 100% purity, 99% yield) as a colorless foam.

LC-MS (Method 2): R_t = 4.72 min; MS (ESIpos): m/z = 1620 $[M+H]^+$.

^{1}H-NMR (400 MHz, DMSO-d6) delta [ppm]: 0.74 - 0.86 (m, 2H), 1.39 (q, 1H), 1.86 - 1.92 (m, 1H), 1.98 - 2.03 (m, 1H), 2.26 - 2.38 (m, 1H), 2.45 (br d, 1H), 2.54 (s, 5H), 2.58 - 2.61 (m, 1H), 2.67 (t, 1H), 2.95 - 3.00 (m, 1H), 3.08 - 3.14 (m, 1H), 3.16 - 3.32 (m, 1H), 3.40 - 3.52 (m, 2H), 3.56 - 3.60 (m, 1H), 3.73 - 3.78 (m, 1H), 4.29 - 4.32 (m, 1H), 5.61 (s, 1H), 6.93 - 6.98 (m, 1H), 7.12 - 7.34 (m, 1H), 7.46 (br s, 1H), 7.77 - 7.81 (m, 1H), 8.38 (s, 1H).

2 Supplementary figures

Figure S1: Rat plasma, human plasma, and buffer pH 7.4 stabilities of SMDCs

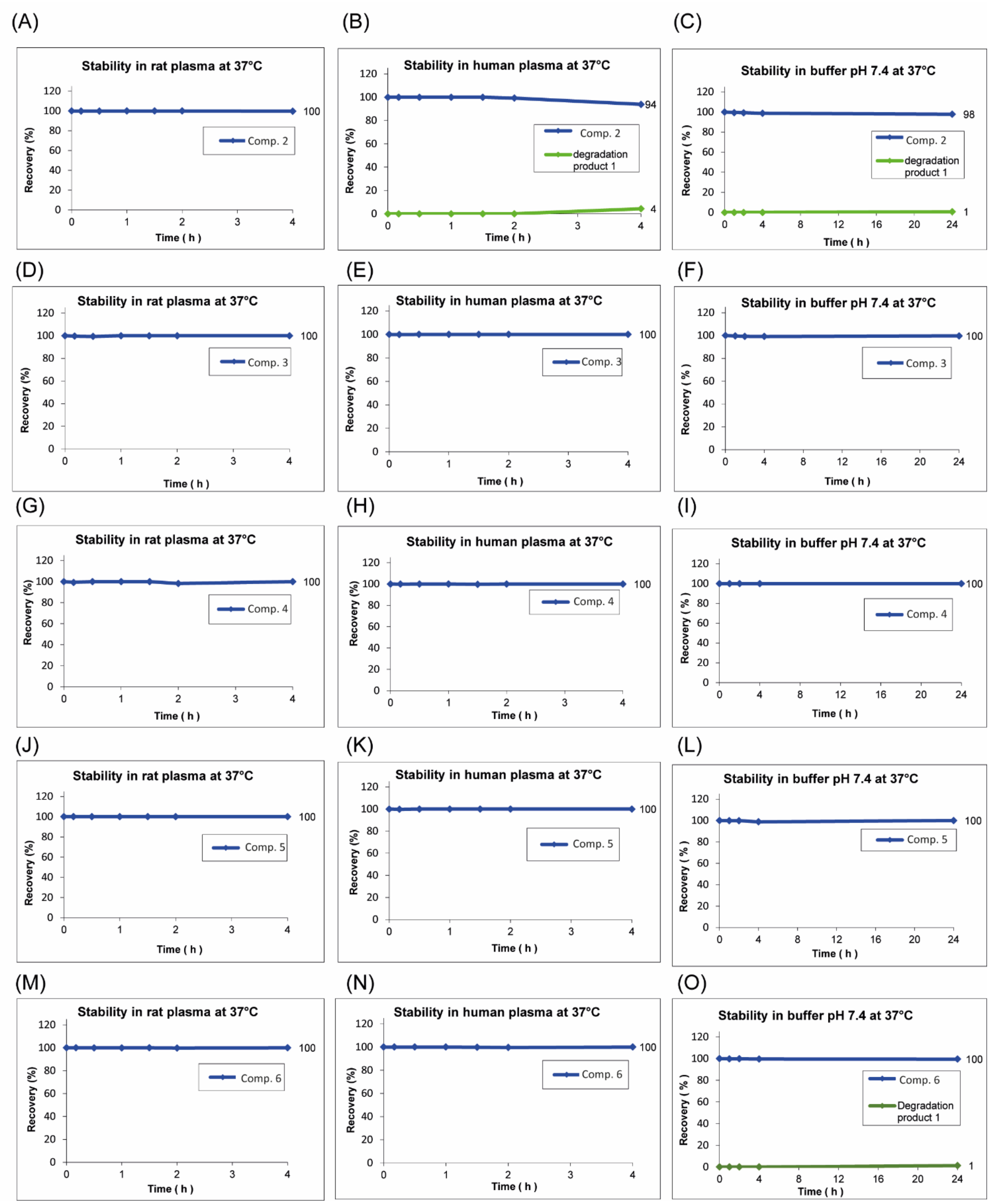

Figure S1: High stability of αvβ3 SMDC conjugates in rat plasma, human plasma and in buffer pH 7.4 at 37C. (A)-(C) Compound 2, (D)-(F) Compound 3, (G)-(I) Compound 4, (J)-(L) Compound 5, (M)-(O) Compound 6. Very small fractions of a degradation product were only detected with Compound 2 in human plasma or in buffer pH7.4 at 37C and with Compound 6 in buffer pH7.4 at 37C.

This is a provisional file, not the final typeset article
